# A general approach to identify low-frequency variants within influenza samples collected during routine surveillance

**DOI:** 10.1099/mgen.0.000867

**Published:** 2022-09-28

**Authors:** Laura A. E. Van Poelvoorde, Thomas Delcourt, Marnik Vuylsteke, Sigrid C. J. De Keersmaecker, Isabelle Thomas, Steven Van Gucht, Xavier Saelens, Nancy Roosens, Kevin Vanneste

**Affiliations:** ^1^​ Transversal activities in Applied Genomics, Sciensano, Juliette Wytsmanstraat 14, Brussels, Belgium; ^2^​ National Influenza Centre, Sciensano, Juliette Wytsmanstraat 14, Brussels, Belgium; ^3^​ Department of Biochemistry and Microbiology, Ghent University, Ghent, Belgium; ^4^​ VIB-UGent Center for Medical Biotechnology, VIB, Ghent, Belgium; ^5^​ Gnomixx, Ghent University, Melle, Belgium

**Keywords:** Influenza, low-frequency variants, next-generation sequencing, patient data, surveillance

## Abstract

Influenza viruses exhibit considerable diversity between hosts. Additionally, different quasispecies can be found within the same host. High-throughput sequencing technologies can be used to sequence a patient-derived virus population at sufficient depths to identify low-frequency variants (LFV) present in a quasispecies, but many challenges remain for reliable LFV detection because of experimental errors introduced during sample preparation and sequencing. High genomic copy numbers and extensive sequencing depths are required to differentiate false positive from real LFV, especially at low allelic frequencies (AFs). This study proposes a general approach for identifying LFV in patient-derived samples obtained during routine surveillance. Firstly, validated thresholds were determined for LFV detection, whilst balancing both the cost and feasibility of reliable LFV detection in clinical samples. Using a genetically well-defined population of influenza A viruses, thresholds of at least 10^4^ genomes per microlitre and AF of ≥5 % were established as detection limits. Secondly, a subset of 59 retained influenza A (H3N2) samples from the 2016–2017 Belgian influenza season was composed. Thirdly, as a proof of concept for the added value of LFV for routine influenza monitoring, potential associations between patient data and whole genome sequencing data were investigated. A significant association was found between a high prevalence of LFV and disease severity. This study provides a general methodology for influenza LFV detection, which can also be adopted by other national influenza reference centres and for other viruses such as SARS-CoV-2. Additionally, this study suggests that the current relevance of LFV for routine influenza surveillance programmes might be undervalued.

## Data Summary

All supporting protocols, code and data have been provided within the article, in the supplementary data or in FigShare: https://doi.org/10.6084/m9.figshare.21214256.v1 [1]. All sequencing reads have been deposited in the NCBI Sequence Read Archive (SRA). All generated consensus genome sequences have been deposited in GISAID.

**Impact Statement**

The influenza virus is prone to mutations and reassortments which leads to a considerable diversity between influenza viruses within different hosts as well as within a host. This results in a population of multiple non-identical viral influenza genomes, or quasispecies, within one patient. Quasispecies may have an impact on the patient by evolving and escaping antiviral drugs, neutralizing antibodies and cytotoxic T-cells. NGS provides the opportunity of not only detecting the majority variant in a sample, but also quasispecies at lower frequencies. This study proposes a general approach to identify low-frequency variants in patient-derived samples obtained during routine surveillance. However, it is quite challenging to distinguish the real low-frequency variants from experimental errors that occur due to PCR and NGS errors. Therefore, validated thresholds were established for low-frequency variant detection, while considering the cost and feasibility of reliable low-frequency variant detection in clinical samples. As a proof of concept for the added value of low-frequency variants for routine influenza monitoring, potential associations between patient data and whole genome sequencing data were investigated.

## Introduction

Influenza is a very contagious respiratory tract infection in humans, mainly caused by the Influenza A and B viruses. Both the Influenza A and B genomes consist of eight segments, including the hemagglutinin (HA) and neuraminidase (NA) segments. Due to their location on the viral envelope, the proteins encoded by the HA and NA segments represent key viral antigens and are the principal targets of the humoral immune response of the host [2–4]. A(H1N1) and A(H3N2) are the two principal Influenza A subtypes that circulate in humans [5].

Influenza viruses have a low-fidelity RNA polymerase that lacks proof-reading functionality. This results in a relatively high mutation rate during viral replication [6]. Replicating influenza within a host does therefore not give rise to genetically identical progeny viruses but rather to ‘quasispecies’, i.e. closely-related viruses that differ by at least one nucleotide from each other. Viral quasispecies are defined as a population of closely-related, non-identical viral genomes in a dynamic host environment that is continuously subjected to competition and selection [7–9]. Although considerable risk exists for producing defective progeny viruses due to the low-fidelity RNA polymerase, this also provides a major opportunity for the virus to rapidly evolve and escape from neutralizing antibodies [10], antiviral drugs [11] and cytotoxic T-cells [12].

The availability and cost-effectiveness of high-throughput sequencing (HTS) technologies have led to their increased use in routine influenza surveillance [13]. HTS allows to determine the sequences of all eight influenza virus segments simultaneously, which offers the opportunity to better understand between- and within-host genetic diversity [14]. Genetic surveillance of influenza virus in biological samples is currently focused on monitoring mutations that are linked to antiviral resistance [15, 16], and antigenic mutations that are relevant for selecting vaccine strains [17]. Studies examining influenza pathogenesis should consequently consider virological and immunological parameters associated to severity as a whole [18]. When investigating viral evolution, transmission, drug and vaccine resistant strains, and pathogenicity, it may not always be sufficient to only examine the consensus genome sequence. Therefore, the current focus is shifting to also include quasispecies while studying genetic diversity [19, 20]. During infection, a particular variant within a quasispecies can by chance obtain a competitive advantage over other variants [21]. This can result in positive selection, and thus an increased frequency of such a variant over time within the patient [22]. However, the spread to other hosts is limited to a small fraction of the quasispecies population and even fewer become fixed in the global viral population [9, 23]. Positive selection of specific quasispecies in hosts has thus far only been observed during long-term infection of immunocompromised patients [24] and in extreme cases of drug resistance [25–27] for the HA and NA genes.

Several recent studies have successfully identified genetic variation in viral quasispecies during clinical influenza infections using deep sequencing with HTS [24, 28–31]. Deep sequencing allows higher genome coverages, and consequently more reliable estimation of the diversity within the quasispecies population present at very low abundances [32]. Apart from the increased experimental costs associated with the use of HTS, many challenges remain to detect low-frequency variants (LFV, i.e. defined as nucleotides differing from the consensus sequence at low allelic frequency at a specific genomic position), including high-quality sequencing reads to ensure that insertions and deletions (indels), and single nucleotide variants (SNVs), can be called confidently. Current variant-calling algorithms for identifying LFV are based on read quality, mapping quality, strand bias, base quality and sequence context [28]. Variants are typically accepted only when their allelic frequency (AF) exceeds the expected sequencing error rate. Several variant-calling methods have been used in multiple HTS-based studies of viral diversity [18, 25, 33]. However, these methods have not always been benchmarked against predefined viral populations, rendering their accuracy for detecting LFV largely unknown. Moreover, not only the bioinformatics approach but also the laboratory process can influence LFV detection. Experimental errors can be introduced during sample preparation, including reverse transcription and PCR amplification, and during sequencing itself [34]. The genome copy number and viral load of samples in particular affect the specificity and sensitivity of variant detection substantially, resulting in more false positive (FP) variant detections for samples with a low concentration due to propagating PCR-amplification errors [28].

In this study, we first established an approach for the quantification of low-frequency variants within influenza samples by using a genetically well-defined population of Influenza A viruses. Thresholds for LFV detection based on HTS with the Illumina technology were validated whilst ensuring that this approach remains powerful enough but also economically feasible in routine surveillance. Secondly, this approach was used to evaluate the prevalence of LFV of influenza A(H3N2) viruses recovered from the Belgian national influenza surveillance network during the 2016–2017 season, demonstrating that several LFV were identified in clinical samples. Finally, potential associations between within-host diversity and patient data were investigated as a proof of concept for the potential relevance of LFV in routine influenza monitoring.

## Methods

### Viruses and cells

A reverse genetics system of Influenza A/Bretagne/7608/2009 (A(H1N1)pdm09) and Influenza A/Centre/1003/2012 (A(H3N2)) in a bidirectional pRF483 plasmid were provided by Institut Pasteur Paris, France. Influenza viruses with a point mutation in the NA segments were obtained by reverse genetics using the QuikChange II Site-Directed Mutagenesis Kit (Agilent Technologies) and GeneJET Plasmid Miniprep Kit (Thermo Fischer) according to the manufacturer’s instructions. For A/Bretagne/7608/2009, the NA-H275Y mutation (CAC → TAT) was introduced (consisting of two nucleotide mutations). For A/Centre/1003/2012, NA-E119V (GAA → GTA) was introduced (consisting of one nucleotide mutation). The NA plasmids were verified using Sanger sequencing on an Applied Biosystems Genetic Analyzer 3500 using the Big Dye Terminator Kit v3.1 following the manufacturer’s instructions using primers described in Table S1.

A co-culture of Madin-Darby canine kidney (MDCK) cells and 293 T cells was maintained in Dulbecco’s modified Eagle medium (DMEM) (Gibco) and 1 % Penicillin Streptomycin (Gibco). The cells were transfected using FuGene HD Transfection Reagent (Promega) and Opti-MEM (Gibco). The viruses were rescued from transfected cells using an 8-plasmid reverse genetic system containing each a genomic segment. Afterwards these viruses were amplified by two cell passages.

### Patient samples

Patient-derived samples were collected from the two main surveillance systems in Belgium, ‘influenza-like-illness’ (ILI) and ‘severe-acute-respiratory-infection’ (SARI). ILI cases are defined by a sudden onset of symptoms, including respiratory and systemic symptoms and fever. A SARI case is defined as an acute respiratory illness with onset within the last 10 days of respiratory symptoms, fever, and requiring hospitalization for at least 24 h. These surveillance systems are in place to follow trends of viral spread and changes in circulating influenza viruses. From these two surveillance systems, initially 253 samples were selected [35, 36]. Only samples with a genome copy number above 10^4^ genomes per microlitre were retained for the LFV validation (see Results), resulting in 59 retained samples, comprising 44 samples from hospitalized SARI patients and 15 from ILI outpatients, spread over the influenza season (beginning, peak and end of epidemic). The genome copy number of 10^4^ genomes per microlitre is based on the Cq values from the routine diagnostic surveillance with qPCR [37] and corresponds with a Cq of 19.53. The samples tested negative using reverse transcription polymerase chain reaction (RT-qPCR) for other respiratory viruses, including respiratory syncytial virus A and B, parainfluenza viruses 1, 2, 3 and 4, enterovirus D68, rhinoviruses, human metapneumoviruses, paraechoviruses, bocaviruses, adenovirus, coronaviruses OC43, NL63, 229 and MERS-CoV [38, 39]. Samples from ILI outpatients were categorized as mild cases (*n*=15). Samples from hospitalized SARI patients were categorized as moderate (*n*=34) or severe cases (*n*=10). Hospital admission (i.e. the SARI case definition) is not a disease severity indicator itself because patients could have been admitted to hospital care for isolation purposes or other medical conditions. A severe case was defined by the presence of at least one severity indicator: death, stay in an intensive care unit, need for invasive respiratory support or extracorporeal membrane oxygenation (ECMO), or the patient having acute respiratory distress syndrome (ARDS). Available patient data are listed in Table 1 with the number of patients exhibiting these characteristics.

**Table 1.** Samples stratified according to patient data

Age (years):	<15	15–59	≥60
**Beginning of epidemic (<week 4**)	4	2	12
**Peak of epidemic (week 4–6**)	2	3	20
**End of epidemic (>week 6**)	4	1	11
**ILI**	15	**SARI**	44
Male*	25	Female*	32
Vaccinated*	11	Not vaccinated*	26
Antibiotics administered*	23	No antibiotics administered*	29
**Respiratory diseases**	9	**No respiratory disease**	50
**Cardiac disease**	18	**No cardiac disease**	41
**Obesity**	6	**No obesity**	53
**Renal insufficiency**	9	**No renal insufficiency**	50
**Diabetes**	6	**No Diabetes**	53
**Immuno-deficiency**	5	**No immuno-deficiency**	54
**Neuromuscular disease**	7	**No neuromuscular disease**	52
**Stay in ICU**	5	**No stay in ICU**	54
Resulting in death*	7	Not resulting in death*	46

*Samples for which certain patient data were unknown, were excluded for analysing that particular characteristic.

Additionally, the median, first quartile and third quartile copy numbers of genomes per microlitre of 1273 A(H3N2) positive influenza samples from the influenza seasons 2015–2019 in Belgium were calculated and plotted with an in-house script (python 3.6) and the matplotlib 3.3.4 library [40] hiding the outliers. The boxplot including the outliers is shown in Fig. S1.

### Creation of mixes of wild-type and mutant viruses

To assess the minimal percentage (i.e. AF) for a LFV to be considered truly present and not constitute a FP observation, mixes were made from the wild-type (WT) and mutant virus, created as described above, for both Influenza A/Bretagne/7608/2009 (A(H1N1)pdm09) and Influenza A/Centre/1003/2012 (A(H3N2)) with eight ratios (0, 0.1, 0.5,1, 5, 10, 20 and 100% mutant virus) (Table S3). Mixes were made in triplicate based on to the plaque forming units (PFU ml^−1^; concentration of virus) of the infectious virus of the WT and mutant. Constructed mixes were situated mainly in the 0–5 % range (Table S4), since previous studies [24, 28–31] have reported most FP being present in this range. RT-ddPCR was used to determine the genome copy numbers of the introduced mutations in the respective mixes (Supplementary Method S2 [1]).

### RNA isolation and RT-qPCR

RNA of the A/Bretagne/7608/2009 (A(H1N1)pdm09) and A/Centre/1003/2012 (A(H3N2)) influenza virus mixes was extracted from culture supernatants using the Easy Mag platform (BioMérieux, #280130-#280134 and #280146) according to the manufacturer’s instructions. Extraction of nucleic acids of clinical specimens was performed using the Viral RNA/DNA isolation kit (Macherey Nagel, Germany, cat No: MN 740691.4). The RNA extraction was done according to manufacturer’s instructions except that the beads were not washed in buffer MV5 but instead left to dry for 10 minutes until the pellet did not appear shiny anymore.

Using 5 µl RNA for each sample, a RT-qPCR was performed using the SuperScriptIII Platinum One-Step Quantitative Kit (Invitrogen) with primers InfA_Forward, InfA_Reverse and InfA_probe. These bind to an influenza M gene section [41]. Each reaction contained 0.5 µl primer/probe, 1 µl SuperScript III RT/Platinum Taq mix, 5 µl nuclease-free water, 12.5 µl PCR Master Mix and 5 µl RNA.

### PCR amplification and whole genome sequencing

To amplify RNA extracts, primers designed to target the 3′ and 5′ conserved ends of all eight segment were used as described previously [35]. Concisely, RT-PCR was used to generate sequencing amplicons in a reaction volume of 50 µl. The used protocol is based on Van den Hoecke *et al*. [32] with optimized volumes and RT-PCR conditions. Primers included CommonA-Uni12G (GCCGGAGCTCTGCAGATATCAGCGAAAGCAGG), CommonA-Uni12 (GCCAGAGCTCTGCAGATATCAGCAAAAGCAGG) and CommonA-Uni13G (GCCGGAGCTCTGCAGATATCAGTAGAAACAAGG) [32]. The reaction volumes included 25 µl RT-PCR buffer, 1 µl SuperScript III One-Step RT-PCR Platinum Taq HiFi DNA Polymerase (Invitrogen, USA), 17.375 µl dH_2_O, 0.375 µl of each primer (20 µM), 0.5 µl RnaseOUT Recombinant Ribonuclease Inhibitor (Invitrogen, USA) and 5 µl of RNA extract. An error rate (number of misincorporated nucleotides per total number of nucleotides polymerized) of lower than 1×10^−3^ by Invitrogen was estimated for the SuperScript III One-Step RT-PCR Platinum Taq HiFi DNA Polymerase [42]. The following PCR conditions were used: one cycle at 42 °C for 15 min, one cycle at 55 °C for 15 min, one cycle at 60 °C for 5 min, one cycle at 94 °C for 2 min (ramp rate: 2.5 °C s^−1^); five cycles at 94 °C for 30 s, 45 °C for 30 s (ramp rate: 2.5 °C s^−1^) and 68 °C for 5 min (ramp rate: 0.5 °C s^−1^); 37 cycles at 94 °C for 30 s, 55 °C for 30 s and 68 °C for 5 min; and one cycle at 68 °C for 5 min (ramp rate: 2.5 °C s^−1^). After purifying the generated amplicons with the NucleoSpin Gel and PCR Clean-up kit (Macherey-Nagel, Germany) according to the manufacturers’ instructions, the concentration of each purification product was quantified with the Qubit 4 Fluorometer (Invitrogen, USA) using the Qubit broad-range assay. Purified products were examined with the Agilent TapeStation (Agilent Technologies, USA) using the Agilent D5000 ScreenTape system.

Sequencing libraries using the Nextera XT DNA Sample Preparation Kit (Illumina, USA) were prepared with the purified RT-PCR products according to the manufacturer’s instructions. All libraries were sequenced on an Illumina MiSeq (Illumina, USA) platform using the MiSeq V3 chemistry, as described by the manufacturer’s protocol, to produce 2×250 bp paired-end reads. Generated WGS data are available in the NCBI Sequence Read Archive (SRA) [43] under accession number PRJNA692424 for the reverse genetics samples (Table S3) and PRJNA615341 for the patient-derived samples (Table S5).

Consensus genome sequences were obtained as described previously [35]. Concisely, using Trimmomatic v0.32 [44], the raw (paired-end) reads were trimmed with the following settings: ‘ILLUMINACLIP:NexteraPE-PE.fa:2 : 30 : 10’, ‘LEADING:10’, ‘TRAILING:10’, ‘SLIDINGWINDOW:4 : 20’, and ‘MINLEN:40’ retaining only paired-end reads. An appropriate reference genome for read mapping was selected from the NCBI viral genomes resource [45] for each sample. Following the GATK ‘best practices’ protocol [46] using Picard v2.8.3 (https://broadinstitute.github.io/picard/) and GATK v3.7, the consensus sequences for all samples were obtained. First, following best practices in the field [47–50], duplicated reads were marked with PICARD MarkDuplicates in order to remove reads originating from PCR duplicates of the same original DNA molecule which could artificially inflate AF of identified variants. This was followed by indel realignment with GATK and variant calling using GATK UnifiedGenotyper with the following options: ‘-ploidy 1’, ‘--stand_call_conf 30’, and ‘--genotype_likelihoods_model BOTH’. Subsequently, only high-quality variants with a read depth ≥200 were retained using GATK VariantFilter. Next, GATK FastaAlternateReferenceMaker was used to obtain the consensus sequence based on the called variants and selected reference sequence.

### Low-frequency variant identification

Only samples with a viral load ≥10^4^ genomes µl^−1^ (see above), and a genome median coverage higher than 1000× calculated as described previously [35], were retained. For LFV calling, the consensus genome fasta files were first indexed using Samtools faidx 1.3.1. Bowtie2-build 2.3.0 [51] was then used to generate indexes. Reads were aligned to the consensus sequence using Bowtie2 align 2.3.0 in end-to-end mode for each sample, producing SAM files that were converted into BAM with Samtools view 1.3.1. Reads were then sorted using Picard SortSam 2.8.3 (http://broadinstitute.github.io/picard/) with the option ‘SORT ORDER=coordinate’. A dictionary of the reference fasta files was created using Picard CreateSequenceDictionary 2.8.3. Reads originating from PCR duplicates which could bias the observed AF of LFV were removed from read alignments using Picard MarkDuplicates 2.8.3 with the option ‘REMOVE_DUPLICATES=true’. The ‘LB’, ‘PL’, ‘PU’ and ‘SM’ flags are required for downstream analysis by GATK and were set to the placeholder value ‘test’ using Picard AddOrReplaceReadGroups 2.8.3. The resulting BAM files were indexed by Samtools index 1.3.1 and used as input for GATK RealignerTargetCreator 3.7 [46] followed by GATK IndelRealigner 3.7 for indel realignment. The generated BAM files were then indexed using Samtools index 1.3.1 and LoFreq 2.1.3.1 [52] was used to detect LFV in ‘call mode’. LoFreq separates true LFV from erroneous variant calls by using Phred-scores as probability error in a Poisson-binomial distribution. The consensus sequence of each sample was used as its own reference to call LFV, in order to avoid calling high-frequency non-reference bases due to an inadequate choice of a single reference sequence for all samples used by LoFreq to call variants, i.e. nucleotides at low allelic frequency differing from the consensus at a specific genomic position [52]. Average read position values were added to called variants using an in-house script (python 3.6) [53] (Supplementary Methods S2 [1]) based on the one provided by McCrone *et al*. [28]. Only variants with a mean reads location within the central 50 % positions (i.e. between bases 62 and 188) were retained for further analysis as advised by McCrone *et al*. [28]. Variants were not further filtered based on Phred-score or mapping quality as was explored in other work, because these metrics are already internally considered by LoFreq for variant calling [28, 52]. An archive containing the code used to call variants and instructions to run it is available as part of the Supplementary Methods S2 [1].

To determine an AF threshold, the workflow described above was used to call variants in the mixes of WT and mutated A(H1N1)pdm09 and A(H3N2) strains. Receiver operating characteristic (ROC) curves for both subtypes were created using an in-house script (python 3.6) and the matplotlib 2.2.2 library [40]. Briefly, called variants were first sorted by decreasing observed AF and then numbers of true and FP variants were calculated at each called AF and plotted as a ROC curve.

### Statistical analysis

All statistical analyses were performed using R-software (RStudio 1.0.153; R3.6.1). Sequencing depth and viral concentration were not introduced as covariates, because we assume that the number of amplification and sequencing errors will be limited due to the validated thresholds set up beforehand (viral concentration=10^4^ copies µl^−1^; allelic frequency=5 % see Results). Furthermore, any remaining amplification and sequencing errors are expected to be distributed randomly over the genome, and these should consequently not have an influence on the statistical analysis. A glm (link function=quasipoisson) was used to assess the association between number of detected LFV and individual patient data parameters, which included disease severity (classified into mild, moderate and severe), patient age, sampling date, sex, vaccination status, presence of comorbidities and disease severity indicators. Patient data were only evaluated if at least 5 % of the retained patient samples met the condition. For example, asthma was not retained because only two out of 59 patients suffered from this condition (3.4 %), whereas vaccination status was retained since 11 out of 59 patients were vaccinated (18.6 %). Afterwards, all identified significant associations (*P* <0.05) were fitted simultaneously in a glm with the same link function and only significant associations were retained. In addition to the median, the interquartile range (IQR) and the effect size were calculated.

## Results

### Validating an AF threshold for LFV calling using an experimental quasispecies population

**Fig. 1.**
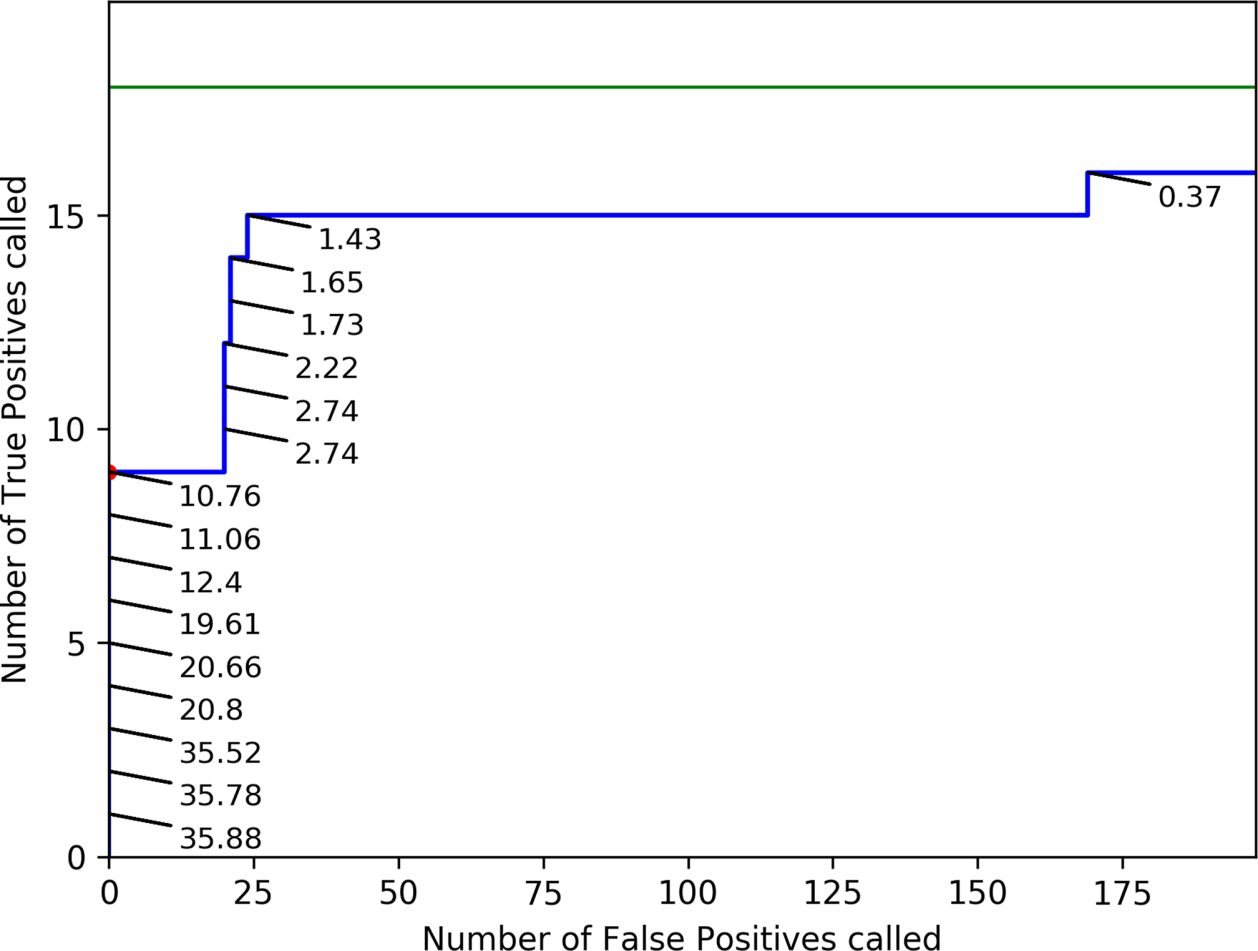
ROC curve for validating an AF threshold using an A(H3N2) benchmark dataset. The green line represents a theoretical scenario where a perfect variant caller identifies all 18 TP before any FP are called (i.e. perfect sensitivity and specificity). The blue line represents the numbers of observed TP and FP in the benchmark dataset for A(H3N2) at decreasing thresholds for the observed AF of called variants. Observed AF of TP are indicated on the graph. AF thresholds used to create the ROC curve are the numbers plotted in the figure (as percentages). The numbers of FP and TP at the threshold of 5 % AF employed for the analysis of patient-derived datasets is depicted by a red dot (no additional TP or FP were observed between 5 and 10.76 %). More detailed values are available in Table 2. AF=Allelic Frequency; ROC=Receiver operating characteristic; FP=False Positive; TP=True Positive.

Sequencing errors affect the frequencies at which variants can reliably be called. At decreasing frequencies, even for high-coverage datasets, the amount of reads containing a certain variant becomes too limited to discriminate real LFV from sequencing errors. Decreasing AF thresholds for accepting LFV will consequently increase sensitivity by identifying more true positive (TP) variants, but also decrease specificity by incorporating more FP variants. It is therefore necessary to establish a validated threshold for the observed AF for accepting LFV. A mutated version of Influenza A/Centre/1003/2012 (A(H3N2)) with high genomic copy number (WT=98 475 genomes µl^−1^; MUT=312 625 genomes µl^−1^) was used to create a validation dataset in triplicate, for which the ground truth was known, to determine an AF threshold for accepting called LFV. The mutant included a specific mutation in the NA segment present at 100 %, i.e. the well-known A(H3N2) oseltamivir resistance mutation NA-E119V [15], which served as a marker when mixing the WT and mutant virus in different ratios (Table S4). The resulting mixes of the eight ratios (theoretically: 0, 0.1, 0.5,1, 5, 10, 20 and 100% mutant virus), and their triplicates, were then subjected to WGS. High sequencing coverages were obtained for all samples and segments (Fig S2), after which LFV were called with LoFreq. Consequently, 18 TP were expected (i.e. one mutation times six ratios (0.1 %, 0.5 %,1 %, 5 %, 10 %, 20 %) times three replicates). Levels of read deduplication were relatively limited (min=21 %, max=61 %, average=36 %; Table S6), and an additional investigation of variants called with and without read deduplication confirmed that read deduplication did not cause any major bias in the numbers of called variants (Supplementary Information S1). Noteworthy, seven additional variants were detected where the mean of the called frequencies over the triplicates corresponded to expected frequencies based on the TP dilution values, as observed at least in one dilution mix with an AF >5 % (Supplementary Information S2). This indicates that during the propagation in cells of both the WT and mutant, other variants emerged even in the absence of external selection pressure. These seven variants were therefore removed from the variant sets used for AF threshold determination as these unexpected variants were not part of the ‘ground truth’, but showed sufficient evidence for being true variants instead of FP (Supplementary Figure S3). Afterwards, TP variants (i.e. the introduced NA mutation in the different mixes) and FP variants (i.e. any variant called in the different mixes that did not correspond with the WT, excluding the seven aforementioned variants) observed at varying observed AFs were expressed in a ROC curve (Fig. 1), considering triplicate values as independent values. The AFs used in the ROC curve are the observed percentages of the NA mutation as determined with Lofreq. A ROC curve expresses the relationship between sensitivity and specificity for a benchmarked experiment where the ground truth is known by varying a discrimination threshold (here the AF) and plotting the false positive rate (i.e. 1-specificity) and sensitivity on the x- and y-axis, respectively. A perfect assay where all FP are separated from TP is characterized by a ROC curve with a right angle that follows the upper left boundary of the plot (Fig. 1).

For A(H3N2), no FP and 50.00 % of TP (*n*=9/18) were called at an observed AF of 4.82 % or higher. This seemingly low sensitivity is explained by the construction of the dataset which aimed at providing a high resolution at low AF to determine the limit of detection and therefore contained half of the variants at an AF lower than 5 %. Decreasing the AF threshold increased the sensitivity but impaired a high cost in specificity (Table 2). At an observed AF of 1%, 83.33 % of TP (*n*=15/18) were recovered at a cost of 289 FP. The highest sensitivity was obtained at an observed AF of 0.37 %, where 88.89 % (*n*=16/18) of variants were called at a cost of 847 FP. An AF cut-off of 5 % was therefore selected as a conservative AF threshold to explicitly minimize the amount of called FP variants to be used for exploring potential associations with host characteristics (see below). Evaluation of the benchmark dataset created for A(H1N1)pdm09 exhibited the same trends, and confirmed 5 % to be an adequate threshold to avoid the inclusion of FP observations (Supplementary Information S3).

**Table 2.** Number of TP, FP, sensitivity, and specificity at different AF thresholds for the A(H3N2) benchmark dataset. Although the specificity remains high due to the size of the negative class (all positions in the genome that are not positives), the number of FP increases dramatically at lower AF, rapidly exceeding more than ten-fold the number of TP. AF=Allelic Frequency; FP=False Positive; TP=True Positive. *: Sensitivity is considered over the full dataset, and not only variants expected at specific AF; see results for further details

Observed AF (%)	no. of TP	no. of FP	Sensitivity (%)*	Specificity (%)
**10.0**	8	0	44.44	100.00
**5.0**	9	0	50.00	100.00
**2.0**	12	86	66.67	99.97
**1.0**	15	289	83.33	99.90
**0.5**	15	678	83.33	99.75

### Selection of patient-derived samples based on their genome copy number

For the described validation of an AF threshold of 5 % based on the experimentally constructed benchmark dataset, all mixes always contained very high genome copy numbers (≥10^5^ genomes µl^−1^, see above). It has been previously established that the genome copy number and titre of samples can also impact LFV calling. Prior research by McCrone *et al*. indicated that samples with a copy number of ≥10^5^ genomes µl^−1^ are acceptable, while samples with a copy number ranging between 10^3^–10^5^ genomes µl^−1^ should be sequenced in duplicate to reduce FP [28]. In routine surveillance, only a limited number of samples however have a copy number of ≥10^5^ genomes µl^−1^. Only 12 out of 253 sequenced samples of the Belgian influenza season 2016–2017 had a genomic copy number ≥10^5^ genomes µl^−1^ (Table S5). This was not due to sample selection bias, since the median of 1273 A(H3N2) positive influenza samples from the influenza seasons 2015–2019 in Belgium was 1168.85 genomes µl^−1^ (IQR: 88.70–8907.89 genomes µl^−1^) (Fig. 1), with a median associated Cq value of 22.52 (IQR: 19.48–26.68), which corresponds to other observations from the literature [54–56].

**Fig. 2.**
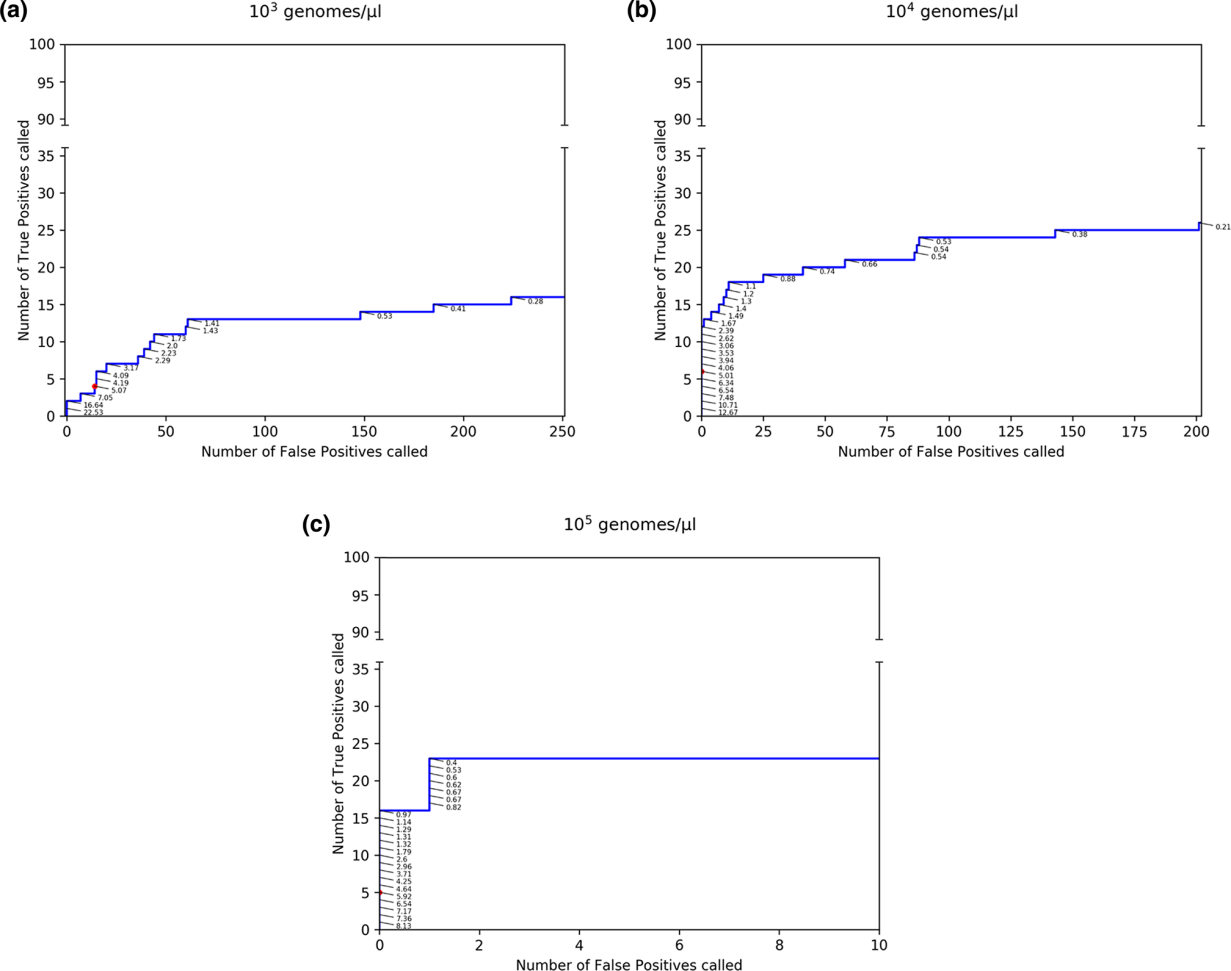
ROC curves to validate an AF threshold using an A(H3N2) benchmark dataset at different genome copy numbers. Observed TP (out of the 100 expected) and FP counts in the benchmark datasets provided by McCrone *et al*. [28] at variable genome copy numbers. The blue line represents observed TP and FP counts in the benchmark dataset for A(H3N2) at variable thresholds for the AF. Observed AF of called TP are plotted in the figure as percentages. The numbers of observed FP and TP at the threshold of 5 % AF employed for the analysis of patient-derived datasets is depicted by a red dot. More detailed values are available in Table 3. Abbreviations: AF=Allelic Frequency; FP=False Positive; TP=True Positive.

To evaluate the impact of adopting a more relaxed genome copy number threshold, we investigated the sensitivity and specificity of the LFV calling workflow on a benchmark dataset containing lower genome copy numbers, for which reference samples of mixes of specific variants at varying targeted AFs and varying initial genomics copy numbers produced and sequenced by McCrone *et al*. [28] were analysed with the same method as described previously. Samples used for this analysis were produced by McCrone *et al*. as an experimental within-host population by inserting 20 mutations in a WSN33 virus genetic background and then diluted to generate five targeted allelic frequencies (5, 2, 1, 0.5 and 0.2 %) and three genomic litres (10^3^, 10^4^ and 10^5^ genomes µl^−1^) [28]. Titres, targeted allelic frequencies and SRA accession numbers of the samples used can be found in Table S2. For samples with 10^3^ genomes µl^−1^, no FP and 2 % of TP (*n*=2/100) were called at an observed AF of ≥16.64 %. These particularly low sensitivities are again the result of the dataset encompassing a majority of low allelic frequency variants. The highest sensitivities, 23, 26 and 16 % for genomic litres of respectively 10^5^, 10^4^ and 10^3^, were obtained at an observed AF of 0.40, 0.21 and 0.28 % at a cost of 1, 201 and 224 called FP, respectively (Fig. 2, Table 3).

**Table 3.** Number of TP, FP, sensitivity, and specificity at different AF thresholds using a A(H3N2) benchmark dataset at different genome copy numbers. Although the specificity remains high due to the size of the negative class (all positions in the genome that are not positives), the number of FP increases dramatically at lower observed AF, an effect which is more pronounced at lower genome copy numbers. *: Sensitivity is considered over the full dataset, and not only variants expected at specific AF; see results for further details

Viral load (genomes µl^−1^)	Observed AF (%)	no. of TP	no. of FP	Sensitivity (%)*	Specificity (%)
**10^5^**	10.0	0	0	0.00	100.00
	5.0	5	0	5.00	100.00
	2.0	10	0	10.00	100.00
	1.0	15	0	15.00	100.00
	0.5	22	1	22.00	99.99
**10^4^**	10.0	2	0	2.00	100.00
	5.0	6	0	6.00	100.00
	2.0	12	0	12.00	100.00
	1.0	18	17	18.00	99.97
	0.5	24	67	24.00	99.90
**10^3^**	10.0	2	1	2.00	99.99
	5.0	4	14	4.00	99.98
	2.0	9	41	9.00	99.87
	1.0	13	83	13.00	99.36
	0.5	14	154	14.00	99.76

Comparison of results for a viral load of ≥10^5^ genomes µl^−1^ of Table 2 and Table 3, indicates similar trends with increasing AF increasing specificity whilst penalizing sensitivity. The sensitivities of the two benchmark datasets in Table 2 and Table 3 are however not directly comparable because the truth set of mutations is present at different AF, resulting in lower sensitivity values for the McCrone dataset because more real variants were present in the observed AF range of 1–5 %. The previously selected AF threshold of 5 % was therefore shown to be a conservative value for filtering out FP variants in datasets obtained from samples with low initial genomic copy numbers because despite removing many TP variants, it also effectively safeguards against including FPs for genome copy numbers at 10^4^–10^5^, but not at 10^3^ genomes µl^−1^. A minimal genome copy number of 10^4^ genomes µl^−1^ was therefore enforced for the clinical dataset.

### Prevalence of LFV in clinical samples

LFV calling was performed on the 59 retained samples with a genome copy number of ≥10^4^ genomes µl^−1^ from the Belgian influenza 2016–2017 A(H3N2) season. When the selected threshold of 5 % AF was used, at least 20 LFV were detected in seven samples, while for 30 samples between 0 and 20 LFV were detected. Finally, 22 samples did not reveal any LFV (Supplementary Method S2 [1]). Across all samples, LFV at 56 genomic positions were detected in two or more patients, including eight located in PB2, six in PB1, 14 in PA, 12 in HA, six in NP, three in NA, one in MP and six in NS. The majority of these variants were detected at a low observed AF of 5–20 %.

### Patient data associated with prevalence of LFV

To investigate the potential relevance of LFV for routine influenza monitoring, a proof of concept investigation based on associations of LFV with patient data was performed. The association of patient data with the number of detected LFV was investigated. After an initial glm analysis where all patient data were evaluated individually, disease severity, antibiotics use and age resulted in a significant association. In a second step, a glm was fitted including the three significant patient data simultaneously, which only resulted into a significant result for disease severity. The number of detected LFV was observed to be significantly higher in ILI cases (i.e. mild cases) compared to SARI cases (i.e. moderate and severe cases) (Table 4; Supplementary Method S2 [1]).

**Table 4.** Statistically significant associations between number of LFV in clinical samples and patient data. Results include the median, first quartile and third quartile of the number of detected LFV across the 59 retained samples, and also *P*-value and effect size. The interpretation of the odds ratio values commonly published in the literature are: <1.68 (small effect), 1.68–3.47 (moderate effect) and >=6.71 (large effect) [70]. ILI cases comprise the mild cases, while the SARI cases include moderate and severe cases. CI=Confidence interval

Patient data	Median	*P*-value	Effect size [CI]
**Disease Severity**	**Mild**: 19 [3.5–60] **Moderate/Severe**: 1 [0–3]	2.67E-08	26.40 [10.89–83.88]

Additionally, associations between patient data and the proportion of nucleotides at their specific genomic positions, including both LFV and high-frequency variants, were evaluated. Although several associations were identified, these were all below acceptable statistical thresholds. These results are therefore provided in the Supplementary Information S4 for informative purposes only and not further considered below.

## Discussion

Since the dynamics of quasispecies can afford influenza a considerable advantage on genetic fitness during within-host evolution, quasispecies information might be relevant for future clinical interventions and epidemiological investigation. HTS renders it nowadays feasible to explore viral quasispecies in patient-derived samples by detecting LFV. However, many challenges remain to obtain reliable results in order to introduce LFV in routine surveillance, in which sampling and funding are often limited. Although HTS enables deep sequencing, it becomes difficult to distinguish sequencing errors from real LFV at low AF. The first goal of this study was to establish an AF threshold for retaining LFV using mixes of a WT and NA-E119V-mutant influenza A(H3N2) virus with different proportions to create a benchmark population that was sequenced followed by LFV calling with LoFreq. While multiple other low-frequency variant callers exist [57–61], LoFreq has been shown to perform particularly well on short read sequencing of virus samples, especially when considering specificity [62, 63]. Other variant callers could alternatively be used as part of the validation approach presented in the current study by other scientists using other software packages. An AF cut-off of 5 % was selected as the minimal AF at which no FP variants were called in the experimentally constructed benchmark A(H3N2) population. An additional exploratory analysis with mixes from the A(H1N1) subtype, which included two nucleotide mutations resulting in the NA-H275Y amino acid mutation, confirmed this as being a robust threshold also applicable to other subtypes (Supplementary Information S3). Since the A(H3N2) and A(H1N1) benchmark populations only contained a single and two nucleotide mutations, respectively, publically available data containing more mutations were also considered. The dataset from McCrone *et al*. includes 20 point mutations and also an extra data point at a theoretical AF of 2%, in contrast to our sequenced A(H3N2) population containing a theoretical AF gap between 1 and 5 %. Analysis of this dataset with our workflow similarly confirmed 5 % to be a robust AF threshold (Fig. 2). This threshold prioritizes specificity over sensitivity, but is context-dependent for three reasons. Firstly, although the established sensitivity of 50 % at 5 % observed AF (Table 2) may appear low, the benchmark dataset was purposefully constructed to assess the limit of detection of our workflow, and therefore contained half of the inserted variants at frequencies lower than 5 %. Conversely, as a result of the choice of thresholds, all variants present at ≥5 % in the benchmark dataset were correctly called. Secondly, since our aim was to evaluate associations of LFV with patient data as a proof of concept, we prioritized specificity to minimize potential FP LFV included within the statistical analysis. Depending on the application scope, this AF threshold can be decreased to increase sensitivity if the cost in specificity is deemed acceptable (e.g. approaches that prioritize finding as many LFV as possible). Thirdly, AF thresholds are coverage-dependent once coverage drops below a certain turnkey point [64], with decreasing coverages typically requiring increased AF thresholds. As both the validation dataset and clinical samples consisted of high-coverage data, our established value of 5 % should only be applied to high-coverage influenza datasets. Through our emphasis on specificity, the selected AF threshold of 5 % is high compared to other AF thresholds reported in other studies in the literature. Gelbart *et al*. [65] investigated the genetic diversity of different viruses, and used a minimum AF threshold of 1 % for highly concentrated samples including human immunodeficiency virus, respiratory syncytial virus, and cytomegalovirus. Orton *et al*. [66] focussed on modelling sequencing errors and distinguishing them from real viral variants using foot-and-mouth disease virus as case study. They established a minimum AF threshold of 0.5%, although this was only tested on control samples that were very highly concentrated (10^6^ plasmid μl^−1^). King *et al*. [67] evaluated laboratory and bioinformatic pipelines to accurately identify LFV in viral populations using foot-and-mouth disease as a case study, using an AF threshold of 0.2 % for highly concentrated samples (10^7^ copies), but observed more errors when a reduced RNA input (10^5^ copies) was used and even found consensus-level errors at (very) low RNA inputs (10^3^ copies).

Previous research has indicated that besides correcting for sequencing errors, the viral load and genome copy number of samples also affect LFV calling, independently of sequencing considerations. In this study, the SuperScript III One-Step RT-PCR Platinum Taq HiFi DNA Polymerase with an estimated error rate of less than 1×10^−3^ misincorporated nucleotides per total number of nucleotides polymerized was used to amplify the virus. This error rate will have a larger impact on samples with low viral loads, because they are more likely to propagate PCR-amplification errors that can result in increased FP variant detections [28]. A genome copy number of 10^5^ genomes µl^−1^ was recommended by McCrone *et al*. and a copy number of 10^3^–10^5^ genomes µl^−1^ was considered acceptable if sequenced in duplicate. However, the application of these recommendations to routine surveillance may prove too restrictive as 10^5^ genomes µl^−1^ is an extremely high copy number for samples encountered in routine influenza surveillance (Fig. S1), where it is already a considerable challenge to acquire the necessary funds to simply switch from Sanger sequencing the HA and NA segments to WGS. As the genome copy number of our experimental dataset was very high (>10^5^ genomes µl^−1^), we employed the experimental within-host population produced by McCrone *et al*. [28] at a genomic input of 10^3^, 10^4^ and 10^5^ genomes µl^−1^ with our workflow to evaluate FP counts at lower genome copy numbers when enforcing the same 5 % AF threshold. We found that also at 10^4^ genomes µl^−1^, no FP were detected, but FP were found at 10^3^ genomes µl^−1^ (Table 3). Similar to our experimentally constructed A(H3N2) benchmark dataset, sensitivities were (very) low because the large majority of LFV were present at AF below 5 %. Notwithstanding, a direct comparison of our results with those reported by McCrone *et al*. is not possible for several reasons. Firstly, McCrone *et al*. used *P*-values as a threshold with either deepSNV or LoFreq to determine effects on sensitivity and specificity in samples of varying targeted AF, whereas we used the observed AF as a threshold with LoFreq with default settings (i.e. *P*-value dynamically adapted as part of a Bonferroni multiple test correction) to determine an AF threshold favouring optimal specificity. Secondly, high specificity at low AF could be obtained by McCrone *et al*. by using deepSNV on both mutated samples and control samples containing the same genetic background. This was initially done with LoFreq on our benchmark datasets using the WT samples as controls and resulted in overall higher specificity and lower sensitivity at very low AF (unpublished results), but does not reflect routine influenza monitoring where no control samples are available for clinical samples to begin with. Thirdly, the samples used by McCrone *et al*. were biassed toward very low AF for the TP, which had a large effect on the sensitivity.

The second goal of this study was to evaluate the prevalence of LFV in actual clinical samples collected during routine influenza monitoring, using 59 influenza A(H3N2) samples from the 2016–2017 Belgian Influenza season with a genome copy number ≥10^4^ genomes µl^−1^ and retaining only LFV detected at ≥5 % AF. It was observed that seven of the 59 samples had at least more than 20 LFV, 30 of the 59 samples had between 0 and 20 LFV, and 22 of the 59 samples did not contain any LFV.

The third goal of this study was to explore potential associations between patient data and the presence and frequency of LFV as a proof of concept for the relevance of LFV analysis in routine influenza surveillance. Statistically significant associations were found between high numbers of LFV and mild cases. It has been suggested in the literature for other viruses that within-host diversity can be driven by host selection pressure [68, 69]. In contrast to our results where more LFV were observed in mild cases, Simon *et al*. observed higher diversity within the PA, HA and NA segments in severe cases compared to mild cases [18]. Additionally, we evaluated potential associations between patient data and the proportion of nucleotides at specific genomic positions. Several associations were found, however, these were below acceptable statistical thresholds (Supplementary Information S4). We are aware, however, of the low statistical power of the association study due to the small sample size of 59 patients and unequal representation of LFV among the patient data groups. More reliable associations will therefore require larger sample sizes in future studies. However, these results show the potential added value to understand viral evolution in relation to the host, but more research is needed.

In conclusion, HTS of clinical influenza samples allows to examine LFV during human infections. Our work provides a general approach for LFV detection by delineating thresholds that balance the number of FP against the feasibility of quasispecies investigation in actual samples collected in the context of routine surveillance programmes. As a proof of concept, several relevant associations with patient data were found while considering LFV, which suggests that the relevance of LFV for influenza monitoring is currently under-valued and could contribute to a better understanding of disease. Although additional validation will be necessary, it could be of great benefit to apply the proposed approach on samples collected during routine influenza monitoring.

## Supplementary Data

Supplementary material 1
