# Supplementary material for: A general approach to identify low-frequency variants within influenza samples collected during routine surveillance
Source: Microb Genom. 2022 Sep 28;8(9):mgen000867. doi: 10.1099/mgen.0.000867 (PMC9676042; doi:10.1099/mgen.0.000867)
Supplement: Supplementary material 1 [file mgen-8-867-s001.pdf]

Table S1: Primers used for Sanger sequencing to verify the sequences of the mutant A(H1N1)pmd09 and A(H3N2) NA-plasmids

**pRF-CMV Fw**	GATAGCGGTTTGACTCACG
**H3N2sNA-2f**	GCAAAAGCAGGAGTAAAGATGAA
**H3N2sNA-418f**	CCTTGGACAGGGAACAAC
**H3N2sNA-550f**	ATGGTCCAGCTCAAGTTGTCA
**H3N2sNA-645R**	CCATCGTAAATGAAGCTAGC
**H3N2sNA-1436r**	CGAAAGCTTATATAGGCATGAGA
**pRF-polyA-Rv**	CTCTAGCATTTAGGTGACC
**H1N1pNA-7f**	AGCAGGAGTTTAAAATGAATCC
**H1N1pNA-459f**	GGAACCATTAAAGACAGGAGC
**H1N1pNA-642r**	ACTTTAACACAGCCACTGC
**H1N1pNA-1070f**	ATTCAAATACGGCAATGGTG
**H1N1pNA-1425r**	TGTCAATGGTAAATGGCAACTC

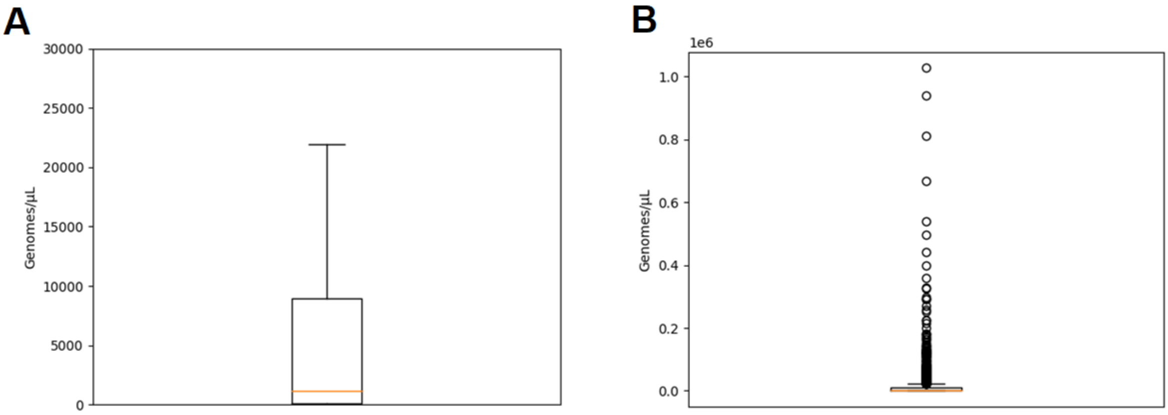

Figure S1: Boxplot of A(H3N2) positive influenza samples from the influenza seasons 2015-2019 in Belgium without (A) and with (B) outliers. The median of 1273 A(H3N2) positive influenza samples was 1168.85 genomes/µL (IQR: 88.70-8907.89 genomes/µL) was 1168.85 genomes/µL (IQR: 88.70–8907.89 genomes/µL).

Table S2: Information on the McCrone samples. This table includes the sample names used internally, SRA accession number, and the genomic titer and the targeted AF of that particular sample.

**Sample name**	**SRA accession**	**Genomic titer (genomes/μl)**	**Targeted AF**
SRR3360142-titer-10-4-freq-0-2	SRR3360142	10^4	0.2
SRR3360143-titer-10-4-freq-0-5	SRR3360143	10^4	0.5
SRR3360144-titer-10-4-freq-1	SRR3360144	10^4	1
SRR3359622-titer-10-4-freq-2	SRR3359622	10^4	2
SRR3359623-titer-10-4-freq-5	SRR3359623	10^4	5
SRR3359624-titer-10-5-freq-0-2	SRR3359624	10^5	0.2
SRR3359625-titer-10-5-freq-0-5	SRR3359625	10^5	0.5
SRR3359626-titer-10-5-freq-1	SRR3359626	10^5	1
SRR3359627-titer-10-5-freq-2	SRR3359627	10^5	2
SRR3359628-titer-10-5-freq-5	SRR3359628	10^5	5
SRR3360149-titer-10-3-freq-0-2	SRR3360149	10^3	0.2
SRR3360150-titer-10-3-freq-0-5	SRR3360150	10^3	0.5
SRR3360151-titer-10-3-freq-1	SRR3360151	10^3	1
SRR3360152-titer-10-3-freq-2	SRR3360152	10^3	2
SRR3360141-titer-10-3-freq-5	SRR3360141	10^3	5

Table S3: Samples of experimental quasi-species for A(H3N2) and A(H1N1)p subtypes and accession numbers. For each sample the subtype, expected WT AF at the mutated positions, proportion of WT and mutated material introduced in the mix, replicate number, Biosample and SRA accession numbers are provided.

Sample_Name	Influenza subtype	Percentage WT	Percentage MUT	Replicate	Biosample accession number	SRA accession number
**1.1_WGS_H1N1p_0_100_Mix1**	H1N1p	0	100	1	SAMN17326320	SRS8037855
**1.2_WGS_H1N1p_0_100_Mix2**	H1N1p	0	100	2	SAMN17326321	SRS8037856
**1.3_WGS_H1N1p_0_100_Mix3**	H1N1p	0	100	3	SAMN17326322	SRS8037867
**1.4_WGS_H1N1p_80_20_Mix1**	H1N1p	80	20	1	SAMN17326323	SRS8037878
**1.5_WGS_H1N1p_80_20_Mix2**	H1N1p	80	20	2	SAMN17326324	SRS8037889
**1.6_WGS_H1N1p_80_20_Mix3**	H1N1p	80	20	3	SAMN17326325	SRS8037898
**1.7_WGS_H1N1p_90_10_Mix1**	H1N1p	90	10	1	SAMN17326326	SRS8037899
**1.8_WGS_H1N1p_90_10_Mix2**	H1N1p	90	10	2	SAMN17326327	SRS8037900
**2.1_WGS_H1N1p_90_10_Mix3**	H1N1p	90	10	3	SAMN17326328	SRS8037901
**2.2_WGS_H1N1p_95_5_Mix1**	H1N1p	95	5	1	SAMN17326329	SRS8037902
**2.3_WGS_H1N1p_95_5_Mix2**	H1N1p	95	5	2	SAMN17326330	SRS8037857
**2.4_WGS_H1N1p_95_5_Mix3**	H1N1p	95	5	3	SAMN17326331	SRS8037858
**2.5_WGS_H1N1p_99_1_Mix1**	H1N1p	99	1	1	SAMN17326332	SRS8037859
**2.6_WGS_H1N1p_99_1_Mix2**	H1N1p	99	1	2	SAMN17326333	SRS8037860
**2.7_WGS_H1N1p_99_1_Mix3**	H1N1p	99	1	3	SAMN17326334	SRS8037861
**2.8_WGS_H1N1p_99.5_0.5_Mix1**	H1N1p	99,5	0,5	1	SAMN17326335	SRS8037862
**3.1_WGS_H1N1p_99.5_0.5_Mix2**	H1N1p	99,5	0,5	2	SAMN17326336	SRS8037863
**3.2_WGS_H1N1p_99.5_0.5_Mix3**	H1N1p	99,5	0,5	3	SAMN17326337	SRS8037864
**3.3_WGS_H1N1p_99.9_0.1_Mix1**	H1N1p	99,9	0,1	1	SAMN17326338	SRS8037865
**3.4_WGS_H1N1p_99.9_0.1_Mix2**	H1N1p	99,9	0,1	2	SAMN17326339	SRS8037866
**3.5_WGS_H1N1p_99.9_0.1_Mix3**	H1N1p	99,9	0,1	3	SAMN17326340	SRS8037868
**3.6_WGS_H1N1p_100_0_Mix1**	H1N1p	100	0	1	SAMN17326341	SRS8037869
**3.7_WGS_H1N1p_100_0_Mix2**	H1N1p	100	0	2	SAMN17326342	SRS8037870
**3.8_WGS_H1N1p_100_0_Mix3**	H1N1p	100	0	3	SAMN17326343	SRS8037871
**4.1_WGS_H3N2_0_100_Mix1**	H3N2	0	100	1	SAMN17326344	SRS8037872
**4.2_WGS_H3N2_0_100_Mix2**	H3N2	0	100	2	SAMN17326345	SRS8037873
**4.3_WGS_H3N2_0_100_Mix3**	H3N2	0	100	3	SAMN17326346	SRS8037874
**4.4_WGS_H3N2_80_20_Mix1**	H3N2	80	20	1	SAMN17326347	SRS8037875
**4.5_WGS_H3N2_80_20_Mix2**	H3N2	80	20	2	SAMN17326348	SRS8037876
**4.6_WGS_H3N2_80_20_Mix3**	H3N2	80	20	3	SAMN17326349	SRS8037877
**4.7_WGS_H3N2_90_10_Mix1**	H3N2	90	10	1	SAMN17326350	SRS8037879
**4.8_WGS_H3N2_90_10_Mix2**	H3N2	90	10	2	SAMN17326351	SRS8037880
**5.1_WGS_H3N2_95_5_Mix1**	H3N2	95	5	1	SAMN17326354	SRS8037883
**5.2_WGS_H3N2_95_5_Mix2**	H3N2	95	5	2	SAMN17326355	SRS8037884
**5.3_WGS_H3N2_95_5_Mix3**	H3N2	95	5	3	SAMN17326353	SRS8037882
**5.4_WGS_H3N2_99_1_Mix1**	H3N2	99	1	1	SAMN17326357	SRS8037886
**5.5_WGS_H3N2_99_1_Mix2**	H3N2	99	1	2	SAMN17326358	SRS8037887
**5.6_WGS_H3N2_99_1_Mix3**	H3N2	99	1	3	SAMN17326356	SRS8037885

**5.7_WGS_H3N2_99.5_0.5_Mix1**	H3N2	99,5	0,5	1	SAMN17326360	SRS8037890
**5.8_WGS_H3N2_99.5_0.5_Mix2**	H3N2	99,5	0,5	2	SAMN17326361	SRS8037891
**6.1_WGS_H3N2_99.5_0.5_Mix3**	H3N2	99,5	0,5	3	SAMN17326359	SRS8037888
**6.2_WGS_H3N2_99.9_0.1_Mix1**	H3N2	99,9	0,1	1	SAMN17326363	SRS8037893
**6.3_WGS_H3N2_99.9_0.1_Mix2**	H3N2	99,9	0,1	2	SAMN17326364	SRS8037894
**6.4_WGS_H3N2_99.9_0.1_Mix3**	H3N2	99,9	0,1	3	SAMN17326362	SRS8037892
**6.5_WGS_H3N2_100_0_Mix1**	H3N2	100	0	1	SAMN17326366	SRS8037896
**6.6_WGS_H3N2_100_0_Mix2**	H3N2	100	0	2	SAMN17326367	SRS8037897
**6.7_WGS_H3N2_100_0_Mix3**	H3N2	100	0	3	SAMN17326365	SRS8037895
**6.8_WGS_H3N2_90_10_Mix3**	H3N2	90	10	3	SAMN17326352	SRS8037881

Table S4: Wild-type (WT) and mutant (MUT) viruses were mixed according to percentages used for the phenotypic assays for A(H1N1)pdm09 and A(H3N2). Values for constructed mixes were verified with ddPCR for A(H3N2).

	WT		MUT	
	**Theoretical**	**ddPCR**	**Theoretical**	**ddPCR**
**Mix 1**	0%	0.34%	100%	99.66%
**Mix 2**	80%	71.20%	20%	28.80%
**Mix 3**	90%	83.39%	10%	16.61%
**Mix 4**	95%	90.94%	5%	9.06%
**Mix 5**	99%	98.11%	1%	1.89%
**Mix 6**	99.5%	99.04%	0.5%	0.96%
**Mix 7**	99.9%	99.76%	0.1%	0.24%
**Mix 8**	100%	99.93%	0%	0.07%

Supplementary Method S1: RT-ddPCR method

The digital droplet PCR was performed using the One-Step RT-ddPCR Advanced Kit for Probes (Bio-Rad) according to the manufacturer's instructions, except that the mix was vortexed for 30 seconds at maximal speed after thawing and between every step the mix was vortexed for 10 seconds at maximal speed. Each reaction had a total volume of 22 µL that was set up on ice, including 0.99 µL of each primer with an initial concentration of 20 µM (H3N2_E119V_FW_Primer 5'-AGGACAATTCGATTAGGCTTTCC-3'; H3N2_E119V_RV_Primer 5'-CTGTCCAAGGGCAAATTGAT-3') and 0.55 µL of each probe with an initial concentration of 10 µM (H3N2_E119_probe FAM-ACA AGA GAA CCT TAT G-MGB-Eclipse; H3N2_V119_probe HEX-ACA AGA GTA CCT TAT G-MGB-Eclipse), 1.1 µL of 300 mM DTT, 8.12 µL of dH_2O, 2.2 µL Reverse Transcriptase, 5.5 µL One-Step Supermix and 2 µL of diluted sample (1:100). According to manufacturer's instructions, 20 µL of the sample mix and 70 µL of Droplet Generation Oil for Probes were loaded into a QX200™ droplet generator (Bio-Rad). After the droplet generation, 40 µL of droplets was recovered per reaction. The RT-PCR amplification reaction was performed in a T100™ Thermal Cycler (Bio-Rad) with the following conditions: one cycle at 50°C for 60 minutes (RT), one cycle at 95°C for 15 minutes (Taq polymerase activation); 40 cycles at 95°C for 30 seconds (denaturation), 55°C for 60 seconds (annealing); and one cycle at 98°C for 10 minutes (enzyme inactivation). Afterwards, the plate was transferred to the QX200 reader (Bio-Rad) and results were acquired using the HEX and FAM channel, according to the manufacturer's instructions. For the interpretation of the results, the QuantaSoft software v1.7.4.0917 (Bio-Rad) was used and the threshold was set manually.

Table S5: List of accession numbers of A(H3N2) dataset and copy number. For each sample the GISAID and SRA accession numbers are given in addition to the copy number.

Isolate_Name	GISAID_Isolate_ID	SRA_Study _Accession	SRA_Sample_ Accession	SRA_Experiment_ Accession	Copy number (genomes/µL)
**A/Belgium/G0007/2017**	EPI_ISL_415204	SRP254509	SRS6396338	SRX8023650	18962.59
**A/Belgium/G0020/2017**	EPI_ISL_415205	SRP254509	SRS6396349	SRX8023661	33709.52
**A/Belgium/G0027/2017**	EPI_ISL_415207	SRP254509	SRS6396411	SRX8023724	78525.22
**A/Belgium/G0051/2017**	EPI_ISL_415215	SRP254509	SRS6396356	SRX8023668	23345.69
**A/Belgium/G0076/2017**	EPI_ISL_415222	SRP254509	SRS6396473	SRX8023785	15190.36
**A/Belgium/G0088/2017**	EPI_ISL_415223	SRP254509	SRS6396318	SRX8023630	32336.33
**A/Belgium/G0125/2017**	EPI_ISL_415228	SRP254509	SRS6396253	SRX8023565	97348.32
**A/Belgium/G0219/2017**	EPI_ISL_415234	SRP254509	SRS6396260	SRX8023572	48337.94
**A/Belgium/G0254/2017**	EPI_ISL_415241	SRP254509	SRS6396269	SRX8023581	16854.76
**A/Belgium/G0369/2017**	EPI_ISL_415260	SRP254509	SRS6396395	SRX8023707	72760.53
**A/Belgium/G0373/2017**	EPI_ISL_415261	SRP254509	SRS6396396	SRX8023708	14672.92
**A/Belgium/G0412/2017**	EPI_ISL_415266	SRP254509	SRS6396403	SRX8023715	66490.86
**A/Belgium/G0437/2017**	EPI_ISL_415272	SRP254509	SRS6396409	SRX8023721	15835.43
**A/Belgium/G0438/2017**	EPI_ISL_415273	SRP254509	SRS6396410	SRX8023722	14173.11
**A/Belgium/G0453/2017**	EPI_ISL_415278	SRP254509	SRS6396437	SRX8023750	22086.37
**A/Belgium/S0021/2017**	EPI_ISL_415292	SRP254509	SRS6396449	SRX8023761	811858.28
**A/Belgium/S0036/2017**	EPI_ISL_415294	SRP254509	SRS6396452	SRX8023763	68360.17
**A/Belgium/S0069/2017**	EPI_ISL_415296	SRP254509	SRS6396453	SRX8023765	1027614.98
**A/Belgium/S0093/2017**	EPI_ISL_415298	SRP254509	SRS6396455	SRX8023767	30380.72
**A/Belgium/S0098/2017**	EPI_ISL_415299	SRP254509	SRS6396270	SRX8023582	75850.36
**A/Belgium/S0150/2017**	EPI_ISL_415305	SRP254509	SRS6396276	SRX8023588	400340.60
**A/Belgium/S0275/2017**	EPI_ISL_415312	SRP254509	SRS6396284	SRX8023596	47343.16
**A/Belgium/S0281/2017**	EPI_ISL_415313	SRP254509	SRS6396285	SRX8023597	18962.59
**A/Belgium/S0365/2017**	EPI_ISL_415320	SRP254509	SRS6396293	SRX8023605	19905.35
**A/Belgium/S0424/2017**	EPI_ISL_415329	SRP254509	SRS6396303	SRX8023615	12597.66
**A/Belgium/S0447/2017**	EPI_ISL_415333	SRP254509	SRS6396307	SRX8023619	23671.58
**A/Belgium/S0457/2017**	EPI_ISL_415338	SRP254509	SRS6396228	SRX8023540	66490.86
**A/Belgium/S0460/2017**	EPI_ISL_415340	SRP254509	SRS6396231	SRX8023543	72760.53
**A/Belgium/S0494/2017**	EPI_ISL_415346	SRP254509	SRS6396237	SRX8023549	21782.30
**A/Belgium/S0499/2017**	EPI_ISL_415347	SRP254509	SRS6396238	SRX8023550	11835.79
**A/Belgium/S0513/2017**	EPI_ISL_415351	SRP254509	SRS6396243	SRX8023555	11197.34
**A/Belgium/S0544/2017**	EPI_ISL_415355	SRP254509	SRS6396247	SRX8023559	14672.92
**A/Belgium/S0549/2017**	EPI_ISL_415356	SRP254509	SRS6396248	SRX8023560	9952.67
**A/Belgium/S0553/2017**	EPI_ISL_415359	SRP254509	SRS6396357	SRX8023669	15402.41
**A/Belgium/S0587/2017**	EPI_ISL_415362	SRP254509	SRS6396360	SRX8023672	19495.70
**A/Belgium/S0724/2017**	EPI_ISL_415371	SRP254509	SRS6396370	SRX8023682	115767.31
**A/Belgium/S0725/2017**	EPI_ISL_415372	SRP254509	SRS6396371	SRX8023683	246438.54
**A/Belgium/S0772/2017**	EPI_ISL_415379	SRP254509	SRS6396379	SRX8023691	138628.89
**A/Belgium/S0785/2017**	EPI_ISL_415383	SRP254509	SRS6396383	SRX8023695	23671.58
**A/Belgium/S0836/2017**	EPI_ISL_415389	SRP254509	SRS6396390	SRX8023702	29549.96
**A/Belgium/S0891/2017**	EPI_ISL_415397	SRP254509	SRS6396419	SRX8023731	228347.01

**A/Belgium/S0896/2017**	EPI_ISL_415399	SRP254509	SRS6396422	SRX8023735	21040.31
**A/Belgium/S0910/2017**	EPI_ISL_415401	SRP254509	SRS6396425	SRX8023737	27955.96
**A/Belgium/S0921/2017**	EPI_ISL_415404	SRP254509	SRS6396428	SRX8023740	10375.32
**A/Belgium/S0931/2017**	EPI_ISL_415405	SRP254509	SRS6396429	SRX8023741	752258.19
**A/Belgium/S0938/2017**	EPI_ISL_415406	SRP254509	SRS6396430	SRX8023742	36128.97
**A/Belgium/S1006/2017**	EPI_ISL_415411	SRP254509	SRS6396436	SRX8023748	45730.48
**A/Belgium/S1029/2017**	EPI_ISL_415413	SRP254509	SRS6396457	SRX8023768	54007.36
**A/Belgium/S1055/2017**	EPI_ISL_415415	SRP254509	SRS6396458	SRX8023770	11672.84
**A/Belgium/S1118/2017**	EPI_ISL_415421	SRP254509	SRS6396465	SRX8023777	52895.90
**A/Belgium/S1166/2017**	EPI_ISL_415424	SRP254509	SRS6396468	SRX8023780	16854.76
**A/Belgium/S1191/2017**	EPI_ISL_415428	SRP254509	SRS6396472	SRX8023784	86527.34
**A/Belgium/S1199/2017**	EPI_ISL_415430	SRP254509	SRS6396474	SRX8023787	137671.31
**A/Belgium/S1247/2017**	EPI_ISL_415433	SRP254509	SRS6396312	SRX8023624	35631.57
**A/Belgium/S1264/2017**	EPI_ISL_415436	SRP254509	SRS6396314	SRX8023626	30170.86
**A/Belgium/S1266/2017**	EPI_ISL_415437	SRP254509	SRS6396315	SRX8023627	100781.30
**A/Belgium/S1268/2017**	EPI_ISL_415438	SRP254509	SRS6396316	SRX8023628	37403.05
**A/Belgium/S1306/2017**	EPI_ISL_415440	SRP254509	SRS6396319	SRX8023631	110284.34
**A/Belgium/S1430/2017**	EPI_ISL_415450	SRP254509	SRS6396330	SRX8023642	297157.53

Table S6: Reads deduplication statistics for all samples (produced during reads deduplication by Picard MarkDuplicates). For each sample the internal name, number of unpaired reads or pair reads examined, number of unpaired reads and read pair marked as duplicates, number of read pair optical duplicates and total percentage of read duplication are provided.

Sample or Isolate Name	Unpaired reads examined	Read pairs examined	Unpaired reads duplicates	Read pairs duplicates	Read pair optical duplicates	Proportion of duplicated reads
**1.1_WGS_H1N1p_0_100_Mix1**	4500	309369	4481	111259	43	0.364225
**1.2_WGS_H1N1p_0_100_Mix2**	5468	487609	5458	199751	46	0.412935
**1.3_WGS_H1N1p_0_100_Mix3**	3824	297716	3802	101308	38	0.344457
**1.4_WGS_H1N1p_80_20_Mix1**	4729	271078	4703	91120	33	0.341832
**1.5_WGS_H1N1p_80_20_Mix2**	3902	290758	3879	102280	27	0.356052
**1.6_WGS_H1N1p_80_20_Mix3**	7941	673629	7930	328598	81	0.490796
**1.7_WGS_H1N1p_90_10_Mix1**	3129	275642	3116	93716	24	0.343693
**1.8_WGS_H1N1p_90_10_Mix2**	3656	237220	3642	73778	28	0.31625
**2.1_WGS_H1N1p_90_10_Mix3**	3281	263228	3266	86350	25	0.332176
**2.2_WGS_H1N1p_95_5_Mix1**	6478	529481	6463	231933	51	0.441441
**2.3_WGS_H1N1p_95_5_Mix2**	7540	528667	7521	229575	80	0.438241
**2.4_WGS_H1N1p_95_5_Mix3**	1866	103974	1834	22024	10	0.218679
**2.5_WGS_H1N1p_99_1_Mix1**	7559	530329	7541	233500	72	0.444237
**2.6_WGS_H1N1p_99_1_Mix2**	1956	100136	1931	20553	14	0.212814
**2.7_WGS_H1N1p_99_1_Mix3**	3201	232247	3181	71638	31	0.313146
**2.8_WGS_H1N1p_99.5_0.5_Mix1**	3958	273650	3939	91808	32	0.340231
**3.1_WGS_H1N1p_99.5_0.5_Mix2**	8189	421314	8168	172589	46	0.415302
**3.2_WGS_H1N1p_99.5_0.5_Mix3**	5772	304751	5748	108477	45	0.361956
**3.3_WGS_H1N1p_99.9_0.1_Mix1**	6559	324930	6539	117287	43	0.367316
**3.4_WGS_H1N1p_99.9_0.1_Mix2**	7557	331309	7532	122707	49	0.377433
**3.5_WGS_H1N1p_99.9_0.1_Mix3**	5647	302236	5623	107686	46	0.362216
**3.6_WGS_H1N1p_100_0_Mix1**	2190	122644	2162	27476	15	0.230784
**3.7_WGS_H1N1p_100_0_Mix2**	3619	201324	3600	60413	28	0.306267
**3.8_WGS_H1N1p_100_0_Mix3**	6440	337671	6418	124044	40	0.373295
**4.1_WGS_H3N2_0_100_Mix1**	2835	252471	2823	85816	24	0.343566
**4.2_WGS_H3N2_0_100_Mix2**	4397	383119	4379	151762	43	0.399545
**4.3_WGS_H3N2_0_100_Mix3**	3984	351611	3969	129777	30	0.372626
**4.4_WGS_H3N2_80_20_Mix1**	6369	449986	6345	193432	43	0.433842
**4.5_WGS_H3N2_80_20_Mix2**	1609	135673	1592	34127	12	0.255888
**4.6_WGS_H3N2_80_20_Mix3**	4009	343139	3993	132255	50	0.388973
**4.7_WGS_H3N2_90_10_Mix1**	2316	256251	2300	88591	34	0.348632
**4.8_WGS_H3N2_90_10_Mix2**	4976	413805	4967	168324	60	0.410306
**5.1_WGS_H3N2_95_5_Mix1**	2583	272160	2568	96555	32	0.357793
**5.2_WGS_H3N2_95_5_Mix2**	3998	251799	3986	88375	28	0.356063
**5.3_WGS_H3N2_95_5_Mix3**	5997	435078	5979	187564	65	0.434978
**5.4_WGS_H3N2_99_1_Mix1**	2674	202600	2655	63671	29	0.318719
**5.5_WGS_H3N2_99_1_Mix2**	3016	280476	3001	101260	32	0.36442

**5.6_WGS_H3N2_99_1_Mix3**	3545	341961	3535	134258	44	0.39573
**5.7_WGS_H3N2_99.5_0.5_Mix1**	1699	181564	1687	54972	13	0.305983
**5.8_WGS_H3N2_99.5_0.5_Mix2**	3224	296595	3209	110498	40	0.375922
**6.1_WGS_H3N2_99.5_0.5_Mix3**	3859	224699	3837	72477	25	0.328271
**6.2_WGS_H3N2_99.9_0.1_Mix1**	4831	395180	4819	162343	44	0.414372
**6.3_WGS_H3N2_99.9_0.1_Mix2**	2794	208482	2778	62478	16	0.304304
**6.4_WGS_H3N2_99.9_0.1_Mix3**	8792	514200	8774	223072	52	0.438605
**6.5_WGS_H3N2_100_0_Mix1**	5285	313362	5268	117308	41	0.379558
**6.6_WGS_H3N2_100_0_Mix2**	2277	159866	2258	42451	16	0.270676
**6.7_WGS_H3N2_100_0_Mix3**	2180	179387	2160	50839	25	0.287677
**6.8_WGS_H3N2_90_10_Mix3**	2804	178785	2786	50493	22	0.287956
**A/Belgium/G0007/2017**	2955	280862	2944	84048	40	0.302898
**A/Belgium/G0020/2017**	4052	226418	4032	72722	26	0.327161
**A/Belgium/G0027/2017**	3439	295058	3418	88498	47	0.303955
**A/Belgium/G0051/2017**	1849	281773	1841	83249	40	0.297737
**A/Belgium/G0076/2017**	3579	268075	3566	77239	38	0.292821
**A/Belgium/G0088/2017**	2674	230566	2665	59001	39	0.260167
**A/Belgium/G0125/2017**	2598	232995	2585	66008	25	0.287248
**A/Belgium/G0219/2017**	3423	254600	3404	74408	27	0.296943
**A/Belgium/G0254/2017**	7732	288137	7703	86980	23	0.311064
**A/Belgium/G0369/2017**	12760	323600	12731	106427	38	0.341816
**A/Belgium/G0373/2017**	4233	265963	4204	82715	29	0.316387
**A/Belgium/G0412/2017**	3982	183146	3950	42760	14	0.241632
**A/Belgium/G0437/2017**	2819	187439	2800	45614	24	0.248951
**A/Belgium/G0438/2017**	4072	427612	4056	166079	53	0.391267
**A/Belgium/G0453/2017**	2496	284421	2481	89191	35	0.31656
**A/Belgium/S0021/2017**	7305	293888	7282	91082	32	0.318353
**A/Belgium/S0036/2017**	8818	334187	8788	114017	44	0.349712
**A/Belgium/S0069/2017**	4644	323824	4626	103804	43	0.325367
**A/Belgium/S0093/2017**	6567	252741	6533	70557	42	0.288345
**A/Belgium/S0098/2017**	6049	388207	6036	130208	48	0.34053
**A/Belgium/S0150/2017**	2628	201476	2606	52479	35	0.26521
**A/Belgium/S0275/2017**	4248	275884	4223	85000	41	0.313342
**A/Belgium/S0281/2017**	7678	476481	7672	184949	71	0.39304
**A/Belgium/S0365/2017**	3481	301741	3470	95691	51	0.321028
**A/Belgium/S0424/2017**	7025	209274	6984	56904	24	0.283834
**A/Belgium/S0447/2017**	3060	252041	3049	72597	45	0.292311
**A/Belgium/S0457/2017**	3548	299845	3526	96133	43	0.324568
**A/Belgium/S0460/2017**	6964	286555	6926	92178	40	0.329754
**A/Belgium/S0494/2017**	6469	294070	6438	96723	29	0.33616
**A/Belgium/S0499/2017**	6343	345135	6321	114296	62	0.337222
**A/Belgium/S0513/2017**	4151	360691	4138	123355	52	0.345743
**A/Belgium/S0544/2017**	4022	296933	4005	95433	49	0.325932
**A/Belgium/S0549/2017**	3942	269068	3919	80212	36	0.303172
**A/Belgium/S0553/2017**	4662	315309	4642	110759	41	0.356001
**A/Belgium/S0587/2017**	6074	286056	6050	85279	40	0.305452

**A/Belgium/S0724/2017**	3280	216687	3256	57264	33	0.269742
**A/Belgium/S0725/2017**	3835	292139	3824	88300	51	0.306785
**A/Belgium/S0772/2017**	3736	262523	3722	76362	39	0.295861
**A/Belgium/S0785/2017**	2741	233080	2723	65180	21	0.283819
**A/Belgium/S0836/2017**	20582	292435	20520	103341	35	0.37526
**A/Belgium/S0891/2017**	5324	263870	5291	80274	35	0.311105
**A/Belgium/S0896/2017**	5833	269884	5810	79955	32	0.303738
**A/Belgium/S0910/2017**	3548	231339	3529	64878	31	0.285881
**A/Belgium/S0921/2017**	2619	282230	2603	86665	42	0.310244
**A/Belgium/S0931/2017**	5883	229421	5852	64089	32	0.288407
**A/Belgium/S0938/2017**	5667	302525	5642	100910	42	0.339702
**A/Belgium/S1006/2017**	4699	291652	4680	89713	47	0.313104
**A/Belgium/S1029/2017**	3385	330832	3376	109081	42	0.333115
**A/Belgium/S1055/2017**	7333	464537	7312	175787	57	0.383259
**A/Belgium/S1118/2017**	5057	332168	5038	105703	43	0.323344
**A/Belgium/S1166/2017**	4019	258111	4001	73289	30	0.289441
**A/Belgium/S1191/2017**	2862	300674	2848	99437	27	0.333861
**A/Belgium/S1199/2017**	10553	242990	10500	72751	40	0.314183
**A/Belgium/S1247/2017**	5104	273013	5089	81410	34	0.304663
**A/Belgium/S1264/2017**	3996	293570	3980	90880	55	0.314209
**A/Belgium/S1266/2017**	3806	303184	3786	94850	40	0.3171
**A/Belgium/S1268/2017**	4077	216707	4055	61532	28	0.290564
**A/Belgium/S1306/2017**	2177	247486	2155	74855	26	0.305472
**A/Belgium/S1430/2017**	7955	243496	7926	68069	27	0.29107
**SRR3359628-titer-10-5-freq-5**	169169	481234	168757	244728	0	0.581647
**SRR3359627-titer-10-5-freq-2**	195524	617377	195177	312756	0	0.573797
**SRR3359626-titer-10-5-freq-1**	173471	558251	173070	197453	0	0.440301
**SRR3359625-titer-10-5-freq-0-5**	193528	537261	193070	248587	0	0.544335
**SRR3359624-titer-10-5-freq-0-2**	184311	561859	183936	271777	0	0.556173
**SRR3359623-titer-10-4-freq-5**	272632	656012	272349	349552	0	0.613037
**SRR3359622-titer-10-4-freq-2**	161235	584577	160863	277214	0	0.537656
**SRR3360144-titer-10-4-freq-1**	254470	821901	254120	417013	0	0.57323
**SRR3360143-titer-10-4-freq-0-5**	175805	584433	175349	275578	0	0.540285
**SRR3360142-titer-10-4-freq-0-2**	140718	456110	140216	208467	0	0.529138
**SRR3360141-titer-10-3-freq-5**	234988	625451	234632	323177	0	0.592901
**SRR3360152-titer-10-3-freq-2**	162102	574014	161693	251803	0	0.507811
**SRR3360151-titer-10-3-freq-1**	280641	818789	280378	379307	0	0.541644
**SRR3360150-titer-10-3-freq-0-5**	223102	652581	222704	277546	0	0.508941
**SRR3360149-titer-10-3-freq-0-2**	246104	746181	245812	290214	0	0.47527

Supplementary Information S1: Evaluation of effects of reads deduplication on called variants counts

PCR duplicate reads occur as a result of the randomness of selection of DNA molecules following PCR amplification prior to sequencing, which results in the same DNA molecule to be sequenced as multiple reads. The AF of called variants based on these reads may therefore not represent the actual AF of the variant in the biological sample, leading to an inflation or decrease in the AF of that variant, which, depending on the filtering method used, may result in the variant being considered or not as a true variant. PCR errors introduced in the amplified DNA may also be picked up as true variant, especially if they appear at an early PCR cycle and therefore are artificially enriched. Duplicate reads identification and removal aims at reducing the bias of representation of specific reads with respect to the original sequence.

The bioinformatics method that was used in the present work (Picard MarkDuplicates [1]) is based on the assumption that reads cover random stretches of the sequenced DNA and therefore should not start and end exactly at the same positions of the genome (using also the position of mate reads as information). Reads presenting the same 5' sequence are considered as putative duplicates, of which the one with maximal base quality score is chosen as representative read, whereas others are marked as duplicates. This process is sequence-agnostic and should therefore not specifically favour mutated or non-mutated reads [2, 3].

Deduplication metrics presented in supplementary table S6 show that between 21-61% of reads were removed during deduplication (average 36%). This does not lead us to believe that a major bias would be observed in the results. To verify this, variants called in deduplicated and non-deduplicated datasets were compared and we observed that 11% of variants called in the deduplicated data were not found in the non-deduplicated call set (523 out of 4934) and 12% of variants called in the non-deduplicated data were not found in the variants from deduplicated data (629 out of 5040). Of the variants that had an AF > 5%, 2.0% of variants called from the deduplicated data were unique to that call set (16 out of 801) and 5.3% of

variants called from the non deduplicated data were unique to that call set (44 out of 829). Three samples accounted for five variants unique to the deduplicated call set and 25 variants unique to the non-deduplicated call set, indicating a potential effect of the samples rather than a systematic bias caused by reads deduplication, especially as these samples were part of the most mutated samples before AF filtering. The majority of variants differentially called in the filtered datasets were called in both sets of the unfiltered datasets, indicating overall a minor variation of the observed AF rather than a major bias in variant calling due to deduplication of the reads. These results indicate that although a noticeable amount of variants were differentially called with and without reads deduplication at AF values below the threshold chosen for inclusion of variants (observed AF = 5%), no strong bias was observed towards one or the other, and noticeably, few variants were called in only the deduplicated or non-deduplicated data.

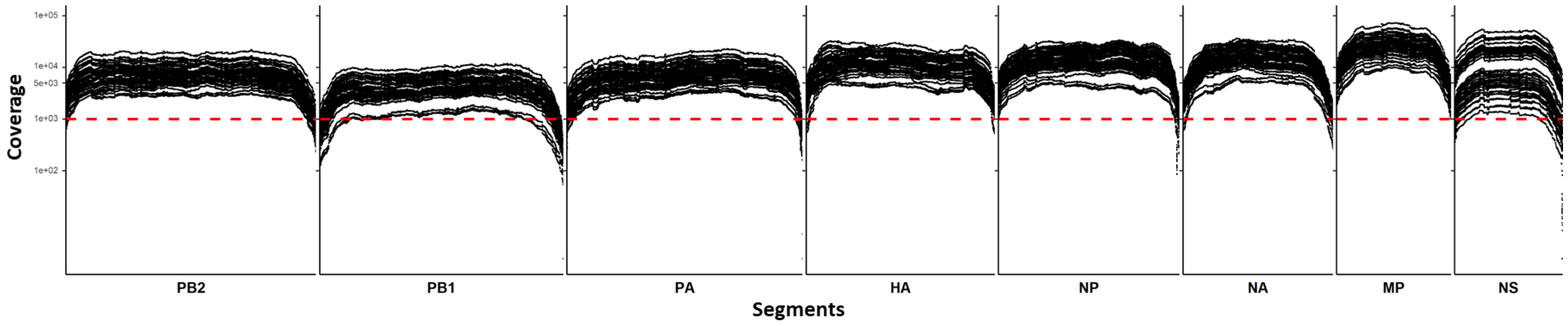

Figure S2: Coverage depth and breadth for the sequenced Influenza A(H3N2) samples using WGS stratified per segment. Coverage depth and gene segment position are presented on the y-axis and x-axis respectively. Every line represents one sample. The red dotted line represents a coverage depth of 1000x.

Supplementary Information S2: Correlation of allelic frequency and mix ratios to discard newly acquired variants

To determine whether unexpected variants of high allelic frequency (AF) were false positives or variants that evolved during virus amplification and should be discarded from the analysis, we evaluate the relation between observed and expected AF by way of a linear regression. Since genomic material was not initial mixed at the perfect ratios, the initial relative abundance of mutated material compared to WT material was estimated based on the observed AF of inserted variants in the material at varying mix ratios. For both virus species (H1N1 and H3N2), the expected AF for each variant at each mix ratio was therefore calculated based on an estimate of the initial mutant relative abundance:

$$\text{exp\_AF} = \frac{\text{mut\_abund} \times \text{ratio}}{((\text{mut\_abund} \times \text{ratio}) + (\text{WT\_abund} \times (1 - \text{ratio}))}$$

where WT_abund was the relative abundance of WT and was equal to 1 and ratio was the mix ratio. The mutant relative abundances were 1.27 and 2.43 for H1N1 and H2N3, respectively and were calculated as the average of mutation abundances calculated for all inserted variants and with an observed AF of at least 5%, as follows:

$$\text{mut\_abund} = \frac{(1 - \text{ratio}) \times \text{obs\_AF}}{(1 - \text{obs\_AF}) \times \text{ratio}}$$

where ratio was the mix ratio and obs_AF was the observed AF of the variant at that mix ratio.

To assess the correlation between expected and observed AF, the coefficient of determination was used, which takes into account both the expected values of AF and the average of all expected values of AF, and thus allows to evaluate the slope of the linear regression. The coefficients of determination (R^2) of observed AF of inserted variants compared to expected AF based on the calculations described above were of 0.95 and 0.99 for H3N2 and H1N1 variants, respectively.

R^2 values above 0.90 were used to remove variants from the datasets of variants to further analyze. In cases where AF of variants could be either above or below 50%, variants were removed from the datasets if at least one of both cases (either variant called or WT) showed $R^2 > 0.5$ and the other case presented a variant AF compatible with the expected AF.

A further analysis on all variants (at high and low AF) identified two variants with a high R^2 value indicating potential TP status, both in H3N2 samples: HA-1040 A-G; NS-645 G-T, with a maximum AF of 2.7% and 2.3% respectively.

Figure S3: Expected and observed AF of unexpected variants of non-low AF in benchmark datasets. (a) Seven variants identified at high frequencies in H3N2 variants call-set and removed from the dataset of variants to analyse based on the high R^2 value. **(b)** Seven variants identified at high frequencies in H1N1 variants call-set and removed from the dataset of variants to analyse based on the high R^2 value.

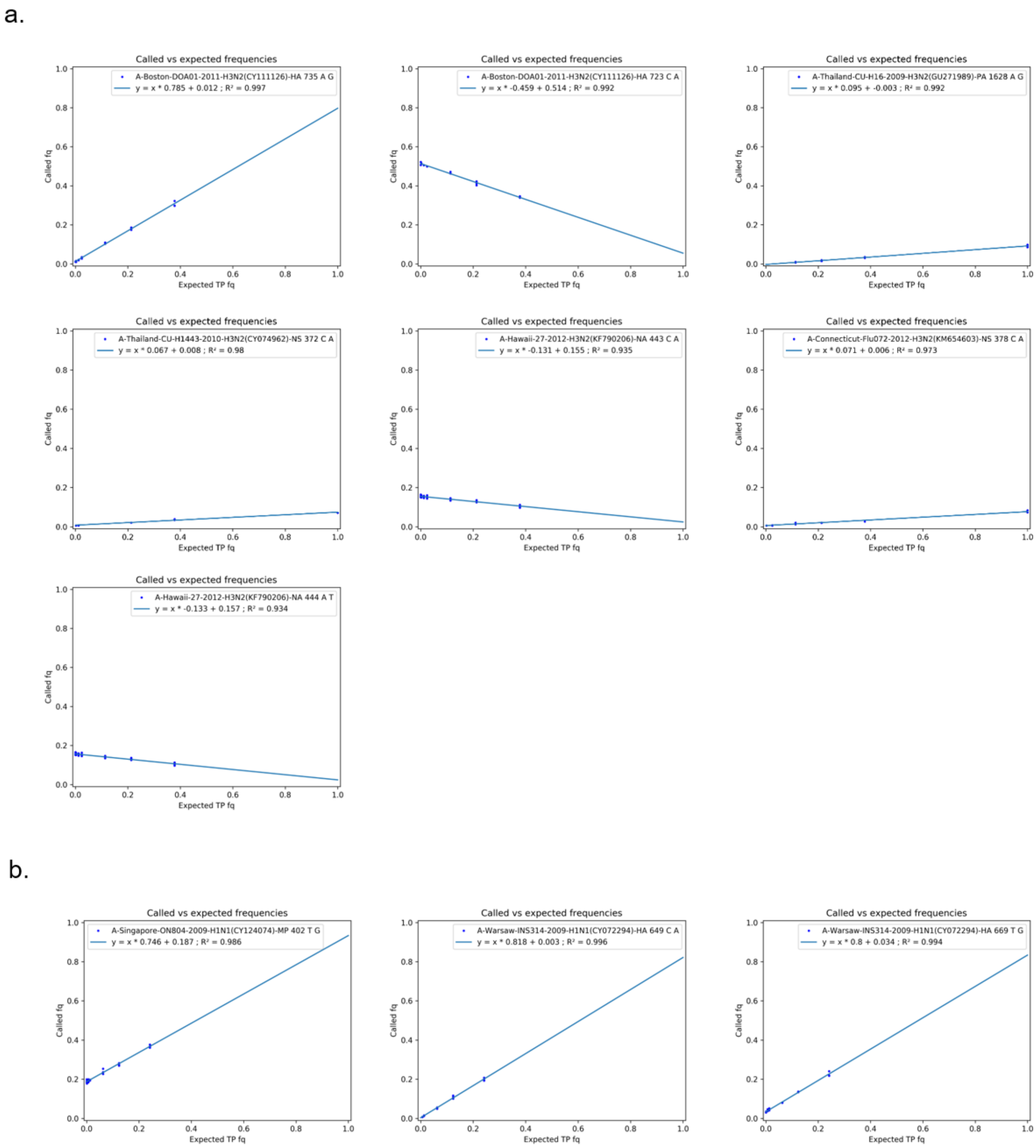
a.
Called vs expected frequencies
A-Boston-DOA01-2011-H3N2(CY111126)-HA 735 A G
y = x * 0.785 + 0.012 ; R² = 0.997
A-Boston-DOA01-2011-H3N2(CY111126)-HA 723 C A
y = x * -0.459 + 0.514 ; R² = 0.992
A-Thailand-CU-H16-2009-H3N2(GU271989)-PA 1628 A G
y = x * 0.095 + -0.003 ; R² = 0.992
A-Thailand-CU-H1443-2010-H3N2(CY074962)-NS 372 C A
y = x * 0.067 + 0.008 ; R² = 0.98
A-Hawaii-27-2012-H3N2(KF790206)-NA 443 C A
y = x * -0.131 + 0.155 ; R² = 0.935
A-Connecticut-Flu072-2012-H3N2(KM654603)-NS 378 C A
y = x * 0.071 + 0.006 ; R² = 0.973
A-Hawaii-27-2012-H3N2(KF790206)-NA 444 A T
y = x * -0.133 + 0.157 ; R² = 0.934
b.
A-Singapore-ON804-2009-H1N1(CY124074)-MP 402 T G
y = x * 0.746 + 0.187 ; R² = 0.986
A-Warsaw-INS314-2009-H1N1(CY072294)-HA 649 C A
y = x * 0.818 + 0.003 ; R² = 0.996
A-Warsaw-INS314-2009-H1N1(CY072294)-HA 669 T G
y = x * 0.8 + 0.034 ; R² = 0.994
Called fq
Expected TP fq

Supplementary Information S3: A(H1N1) results

An additional exploratory analysis was performed to evaluate if this 5% threshold could be applicable to another influenza A virus subtype. This was done using the influenza virus A/Bretagne/7608/2009 (A(H1N1)pdm09) (WT=1,378,182 copies/µL; MUT=1,716,364 copies/µL). A dataset was created in triplicate, for which the ground truth was known, to validate a threshold for employed AF. The mutant included a specific mutation in the NA segment present at 100%, namely the well-known oseltamivir resistance mutation H275Y in the NA of A(H1N1)pdm09 virus [4], which served as markers when mixing the wild-type and mutant virus in different ratios. The presence of the mutation in the mutant virus was confirmed by conventional Sanger sequencing of the NA gene. The resulting mixes, and their triplicates, were then subjected to WGS, obtaining high coverage for all segments (see Supplementary Figure S1, supplementary Table S4), after which low-frequency variants were called with LoFreq. For A(H1N1)pdm09, no false positives (FP) and 50.00% of true positives (TP) were called at an AF of 3.04%. Decreasing the AF to obtain higher sensitivity immediately impaired a high cost in sensitivity. At an AF of 1%, 66.67% of TP (24/36) were recovered at a cost of 66 FP for A(H1N1)pdm09. Maximum sensitivity (83.34%, 30/36) was obtained at an AF of 0.61%, but at the cost of 122 FP. This analysis, combined with the one of H3N2 sequencings, suggests that 5% is a stable and conservative cut-off (Supplementary Figure S4; Supplementary Table S7).

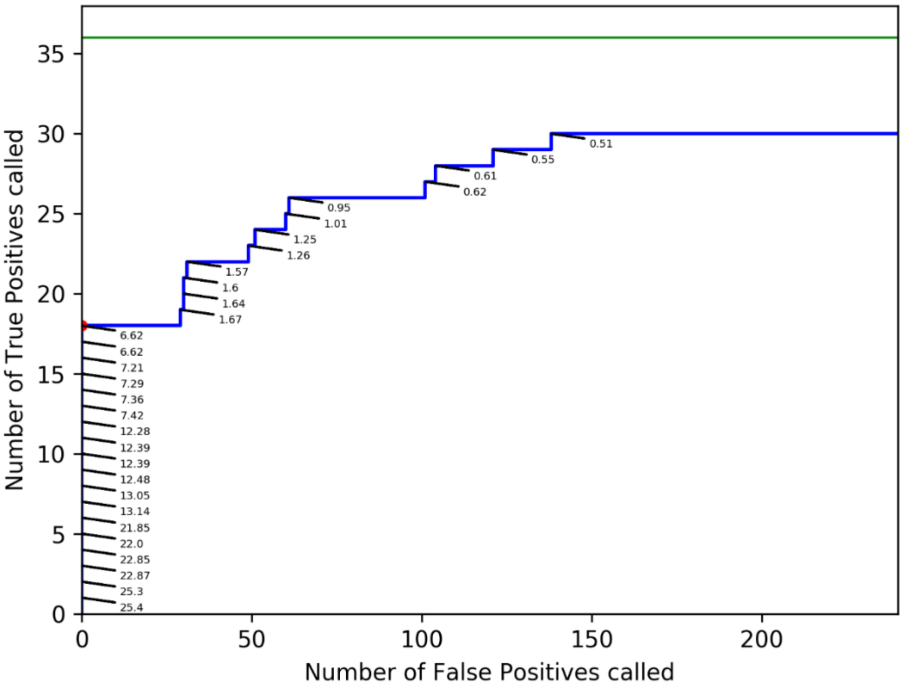

Figure S4: ROC curve for A(H1N1)pdm09 strain. The green **curve** represents a theoretical scenario where a perfect variant caller identifies all 36 true positives before any false positive are called (i.e. perfect sensitivity and specificity). The blue curve represents observed true positive and false positive counts in the benchmark datasets for A(H1N1)pdm09 at variable thresholds for the AF at which variants were accepted. AF thresholds used to create the ROC curve (AF of each true positive) are plotted in the figure. For A(H1N1)pdm09, no false positives and 50.00% of positives (18/36) were called at an AF of 6.62%. The first FP was called at a frequency of 2.86%. The numbers of TP and FP expected when employing an AF threshold of 5% (respectively 18 and none) is depicted by a red dot.

Table S7: Performance of the workflow at varying allelic frequency thresholds. True positive counts, False positive counts, sensitivity and specificity of the workflow applied to the H1N1 datasets when using varying allelic frequency thresholds. As was observed for the H3N2 datasets, reducing the AF threshold increases the sensitivity and negatively affects the specificity. Again, although the specificity remains high due to the size of the negative class (all positions in the genome which are not positives), the number of False positives increases dramatically at lower AF, although exceeding the number of TP less than for the H3N2 datasets.

AF (%)	Number of TP	Number of FP	Sensitivity (%)	Specificity (%)
**10.0**	11	0	33.56	100.00
**5.0**	17	0	47.22	100.00
**2.0**	18	19	50.00	99.99
**1.0**	24	60	66.67	99.98
**0.5**	30	138	83.33	99.95

Supplementary Information S4: Individual nucleotide analysis

Methods

A glm (link function = binomial) was used to assess the association between particular nucleotides at specific genomic positions with their associated frequency and patient data. Only positions where at least 10% of samples exhibited the presence of certain LFV, as well as the high-frequency variants (HFV), were retained. Positions where no variation between samples was observed (i.e. nucleotide was present at 0% or 100% at a specific position for all samples) were excluded from the analysis. Since fewer significant results than expected under the False Discovery Rate (FDR) null-hypothesis of uniformly distributed p-values were obtained, the real significances could not be modelled. Consequently, a more stringent p-value threshold of 0.01 was applied to reduce the number of false positive results. Given the 379 nucleotides at certain positions that were interrogated for association with a clinical parameter, we only report clinical parameters having more significant associations than the four that are expected by chance at the alpha-level of 0.01. Additionally, the effect size was calculated. All analysis scripts and results are provided in Supplementary Method S2.

Results

Associations between the host characteristics and the proportion of nucleotides at their specific genomic positions, including both LFV and HFV, were evaluated. Several statistically significant associations were found (Table S3; Supplementary Method S2), including sample period and the presence of obesity in the patient. In all cases, two significant associations were detected at the same position with a different nucleotide. One of the two nucleotides is then linked to the patient data (i.e., obesity, or sampling period), while the other nucleotide is linked with to the reverse condition for that characteristic. These two different nucleotides at the same position always result in synonymous mutations except for the significant associations observed with the sampling date at HA-411 and NA-418. Table S3 indicates that 32 LFV were detected at different 16 genomic positions associated with patient data, in addition to the HFV variants and consensus nucleotide detected at that position.

Table S3: Statistically significant associations between nucleotides at specific genomic positions and patient data (considering both LFV and HFV). Results include a description of the functional site of the position, the number of samples that show a LFV (i.e. <50%, excluding 0%), HFV (i.e. ≥50%, excluding 100%) or how many samples that have the nucleotide in the consensus sequence (#CON) (i.e. 100%) at each position for each group of the patient data versus the total number of patients that were included in that respective group of the patient data, the p-value, and the odds ratio. The LFV are the results of the LFV detection approach, while the HFV include the result of subtracting the initial 100% consensus nucleotide by the result of the LFV detection. Most amino acids remain the same at the positions of interest. Positions for which the amino acid changes are highlighted in yellow. The sampling periods are divided in "Beginning", "Peak", and "End". The interpretation of the odds ratio values commonly published in the literature are: <1.68 (small effect), 1.68 - 3.47 (moderate effect) and >= 6.71 (large effect) **[5]**.LFV=Low-frequency variant; HFV=High-frequency variant; AA=Amino acid; CON=Consensus; CI=Confidence interval

Patient data	Segment	Nucleotide (AA)	Functional site	Associated with	Number of samples per characteristic					P-value	Odds Ratio [CI]
					Patient data	#LFV	#HFV	#CON	#Total		
Obesity	NA	A1095 (T365)	Catalytic domain [6–11]	Not obese	Not obese<br>Obese	0<br>1	1<br>0	48<br>2	53<br>6	0.002	21.03 [3.21; 180.60]
		G1095 (T365)		Obese	Not obese<br>Obese	1<br>0	0<br>1	4<br>3	43<br>6	0.002	
	NP	A510 (S170)	Not described	Obese	Not obese<br>Obese	1<br>0	1<br>1	6<br>3	53<br>6	0.008	12.01 [1.99; 94.35]
		G510 (S170)		Not obese	Not obese<br>Obese	1<br>1	1<br>0	45<br>2	53<br>6	0.008	
		C1191 (N397)	Overlapping NP and PB2 interaction region [12]	Obese	Not obese<br>Obese	1<br>0	0<br>1	5<br>3	53<br>6	0.003	16.31 [2.60; 133.51]
		T1191 (N397)		Not obese	Not obese<br>Obese	0<br>1	1<br>0	47<br>2	53<br>6	0.003	
	PA	C135 (C45)	Endonuclease activity [13, 14]	Obese	Not obese<br>Obese	1<br>0	0<br>1	6<br>3	53<br>6	0.005	13.95 [2.27; 111.87]
		T135 (C45)		Not obese	Not obese<br>Obese	0<br>1	1<br>0	46<br>2	53<br>6	0.005	
		C978 (H326)	Not described	Not obese	Not obese<br>Obese	1<br>1	1<br>0	45<br>2	53<br>6	0.008	11.79 [1.95; 91.63]
		T978 (H326)		Obese	Not obese<br>Obese	1<br>0	1<br>1	6<br>3	53<br>6	0.008	
		A1056 (E352)	Not described	Obese	Not obese<br>Obese	1<br>0	0<br>1	4<br>3	53<br>6	0.002	20.98 [3.21; 180.13]
		G1056 (E352)		Not obese	Not obese<br>Obese	0<br>1	1<br>0	48<br>2	53<br>6	0.002	

	PB1	C915 (D305)	Not described	Not obese	Not obese<br>Obese	0<br>1	1<br>0	48<br>2	53<br>6	0.002	21.26 [3.25; 183.85]	
		T915 (D305)		Obese	Not obese<br>Obese	1<br>0	0<br>1	4<br>3	53<br>6	0.002		
		C1293 (Y431)	Not described	Not obese	Not obese<br>Obese	0<br>1	1<br>0	48<br>2	53<br>6	0.002	21.46 [3.28; 186.23]	
		T1293 (Y431)		Obese	Not obese<br>Obese	1<br>0	0<br>1	4<br>3	53<br>6	0.002		
**Sampling period**	HA	A411 (K137)	Epitope A [15]	End	Beginning<br>End	2<br>0	0<br>1	3<br>10	18<br>16	0.007	Beginning vs Peak<br>Beginning vs End<br>Peak vs End	4.83 [1.25; 23.24]<br>1.91 [.53; 7.50]<br>9.24 [2.07; 52.09]
		C411 (N137)		Beginning	Beginning<br>End	0<br>1	2<br>0	13<br>5	18<br>16	0.007		
	NA	A418 (I140)	Catalytic domain [6–11]	Beginning	Beginning<br>Peak/End	0<br>0	1<br>0	13<br>15	18<br>41	0.007	Beginning vs Peak<br>Beginning vs End<br>Peak vs End	5.75 [1.57; 24.73]<br>1.08 [.29; 3.96]<br>5.33 [1.28; 25.90]
		C418 (L140)		Peak & End	Beginning<br>Peak/End	1<br>0	0<br>0	4<br>26	18<br>41	0.007		
	NP	A531 (G177)	Not described	Beginning	Beginning<br>Peak/End	2<br>1	0<br>1	4<br>25	18<br>41	0.007	Beginning vs Peak<br>Beginning vs End<br>Peak vs End	5.63 [1.54; 24.05]<br>1.08 [.28; 3.95]<br>5.23 [1.26; 25.26]
		G531 (G177)		Peak & End	Beginning<br>Peak/End	0<br>1	2<br>1	12<br>14	18<br>41	0.007		
		A1152 (G384)	Overlapping NP and PB2 interaction region [12]	Peak & End	Beginning<br>Peak/End	0<br>1	2<br>1	12<br>14	18<br>41	0.007	Beginning vs Peak<br>Beginning vs End<br>Peak vs End	5.61 [1.54; 23.93]<br>1.08 [.29; 3.97]<br>5.19 [1.25; 25.04]
		G1152 (G384)		Beginning	Beginning<br>Peak/End	2<br>1	0<br>1	4<br>25	18<br>41	0.007		
	PA	A837 (R279)	PA linker and external linker[16]	Beginning	Beginning<br>Peak/End	1<br>1	0<br>2	4<br>25	18<br>41	0.004	Beginning vs Peak<br>Beginning vs End<br>Peak vs End	5.62 [1.54; 24.01]<br>1.17 [.31; 4.62]<br>6.57 [1.56; 32.79]
		G837 (R279)		Peak & End	Beginning<br>Peak/End	0<br>2	1<br>1	13<br>13	18<br>41	0.004		
		C1320 (S440)	PB1-binding domain [16]	Peak & End	Beginning<br>Peak/End	3<br>1	0<br>1	4<br>26	18<br>41	0.005	Beginning vs Peak<br>Beginning vs End<br>Peak vs End	5.57 [1.53; 23.70]<br>1.17 [.31; 4.64]<br>6.52 [1.55; 32.47]
		T1320 (S440)		Beginning	Beginning<br>Peak/End	0<br>1	3<br>1	11<br>13	18<br>41	0.005		
	PB1	C141 (H47)	Not described	Peak & End	Beginning<br>Peak/End	0<br>0	0<br>1	4<br>25	18<br>41	0.006	Beginning vs Peak<br>Beginning vs End<br>Peak vs End	6.17 [1.66; 27.25]<br>1.14 [.30; 4.17]<br>5.42 [1.29; 26.79]
		T141 (H47)		Beginning	Beginning<br>Peak/End	0<br>1	0<br>0	14<br>15	18<br>41	0.006		
		A1785 (G595)	Not described	Peak & End	Beginning<br>Peak/End	2<br>1	0<br>1	4<br>26	18<br>41	0.005	Beginning vs Peak<br>Beginning vs End<br>Peak vs End	5.58 [1.53; 23.73]<br>1.14 [.31; 4.49]<br>6.36 [1.51; 31.55]
		G1785 (G595)		Beginning	Beginning<br>Peak/End	0<br>1	2<br>1	12<br>13	18<br>41	0.005		

References

1. **Broad Institute**. Picard Tools. https://broadinstitute.github.io/picard/ (2009, accessed 24 September 2021).

2. **GATK**. MarkDuplicates (Picard). *GATK*. https://gatk.broadinstitute.org/hc/en-us/articles/360037052812-MarkDuplicates-Picard- (2021, accessed 27 January 2022).

3. **Marx V**. How to deduplicate PCR. *Nat Methods* 2017;14:473–476.

4. **Boivin G**. Detection and management of antiviral resistance for influenza viruses. *Influenza and other Respiratory Viruses* 2013;7:18–23.

5. **Chen H, Cohen P, Chen S**. How Big is a Big Odds Ratio? Interpreting the Magnitudes of Odds Ratios in Epidemiological Studies. *Communications in Statistics - Simulation and Computation* 2010;39:860–864.

6. **Hirst GK**. ADSORPTION OF INFLUENZA HEMAGGLUTININS AND VIRUS BY RED BLOOD CELLS. *The Journal of experimental medicine* 1942;76:195–209.

7. **Gottschalk A**. Neuraminidase: the specific enzyme of influenza virus and Vibrio cholerae. *Biochimica et Biophysica Acta* 1957;23:645–646.

8. **Palese P, Compans RW**. Inhibition of Influenza Virus Replication in Tissue Culture by 2-deoxy-2,3-dehydro-N-trifluoroacetylneuraminic acid (FANA): Mechanism of Action. *Journal of General Virology* 1976;33:159–163.

9. **Russell RJ, Haire LF, Stevens DJ, Collins PJ, Lin YP, *et al.*** The structure of H5N1 avian influenza neuraminidase suggests new opportunities for drug design. *Nature* 2006;443:45–49.

10. **Webster RG, Laver WG**. Preparation and properties of antibody directed specifically against the neuraminidase of influenza virus. *Journal of immunology (Baltimore, Md : 1950)* 1967;99:49–55.

11. **Varghese JN, Laver WG, Colman PM**. Structure of the influenza virus glycoprotein antigen neuraminidase at 2.9 Å resolution. *Nature* 1983;303:35–40.

12. **Ping J, Keleta L, Forbes NE, Dankar S, Stecho W, *et al.*** Genomic and Protein Structural Maps of Adaptive Evolution of Human Influenza A Virus to Increased Virulence in the Mouse. *PLoS ONE* 2011;6:e21740.

13. **Dias A, Bouvier D, Crépin T, McCarthy AA, Hart DJ, *et al.*** The cap-snatching endonuclease of influenza virus polymerase resides in the PA subunit. *Nature* 2009;458:914–918.

14. **Yuan P, Bartlam M, Lou Z, Chen S, Zhou J, *et al.*** Crystal structure of an avian influenza polymerase PAN reveals an endonuclease active site. *Nature* 2009;458:909–913.

15. **Jorquera PA, Mishin VP, Chesnokov A, Nguyen HT, Mann B, *et al.*** Insights into the antigenic advancement of influenza A(H3N2) viruses, 2011–2018. *Scientific Reports* 2019;9:2676.

16. **Wang L, Wu A, Wang YE, Quanquin N, Li C, *et al.*** Functional Genomics Reveals Linkers Critical for Influenza Virus Polymerase. *Journal of Virology* 2016;90:2938–2947.
